# Mutant-selective degradation by BRAF-targeting PROTACs

**DOI:** 10.1038/s41467-021-21159-7

**Published:** 2021-02-10

**Authors:** Shanique Alabi, Saul Jaime-Figueroa, Zhan Yao, Yijun Gao, John Hines, Kusal T. G. Samarasinghe, Lea Vogt, Neal Rosen, Craig M. Crews

**Affiliations:** 1 Department of Pharmacology, New Haven, CT USA; 2 Molecular, Cellular, and Developmental Biology, Yale University, New Haven, CT USA; 3 Program in Molecular Pharmacology, Memorial Sloan Kettering Cancer Center, New York, NY USA; 4 Department of Chemistry, Yale University, New Haven, CT USA

**Keywords:** Oncogene proteins, Proteolysis, Targeted therapies, Chemical tools

## Abstract

Over 300 BRAF missense mutations have been identified in patients, yet currently approved drugs target V600 mutants alone. Moreover, acquired resistance inevitably emerges, primarily due to RAF lesions that prevent inhibition of BRAF V600 with current treatments. Therefore, there is a need for new therapies that target other mechanisms of activated BRAF. In this study, we use the Proteolysis Targeting Chimera (PROTAC) technology, which promotes ubiquitination and degradation of neo-substrates, to address the limitations of BRAF inhibitor-based therapies. Using vemurafenib-based PROTACs, we achieve low  nanomolar degradation of all classes of BRAF mutants, but spare degradation of WT RAF family members. Our lead PROTAC outperforms vemurafenib in inhibiting cancer cell growth and shows in vivo efficacy in a Class 2 BRAF xenograft model. Mechanistic studies reveal that BRAF^WT^ is spared due to weak ternary complex formation in cells owing to its quiescent inactivated conformation, and activation of BRAF^WT^ sensitizes it to degradation. This study highlights the degree of selectivity achievable with degradation-based approaches by targeting mutant BRAF-driven cancers while sparing BRAF^WT^, providing an anti-tumor drug modality that expands the therapeutic window.

## Abstract (web summary)

Hundreds of BRAF mutations have been identified in patients with cancer but currently approved drugs only target BRAF V600 mutants. Here, the authors develop a vemurafenib-based PROTAC that induces degradation of all classes of BRAF mutants without affecting wild-type RAF proteins.

## Introduction

The Ras-RAF-MEK-ERK pathway is important for many aspects of cellular homeostasis^1^. The pathway is initiated upon extracellular growth factor binding to receptor tyrosine kinases (RTKs), thereby activating the kinase cascade^2^. Upon activation of upstream effectors, GTP-bound RAS recruits RAF (ARAF, BRAF, or CRAF) to the cell membrane, promoting its dimerization and activation^2^. Thus, the scaffolding and enzymatic role of BRAF are both essential for its function^3–5^. Activated RAF phosphorylates and activates MEK, which in turn phosphorylates and activates ERK leading to cell proliferation, differentiation, and survival^2^. BRAF is mutated in 8% of observed cancers including melanoma (60%)^6^, colorectal cancer (10%)^7^, non-small cell lung cancer (NSCLC) (10%)^8^, and hairy cell leukemia (100%)^9^. These mutations (often missense mutations found in the kinase domain) distinctly affect the biochemical characteristics of the kinase^10–12^. Class 1 BRAF mutants such as V600E and V600K are hyper-activating and can signal as monomers in the absence of activated RAS^13^. Class 2 BRAF mutants such as K601E and G469A signal as constitutive, RAS- independent dimers^14^. Lastly, Class 3 BRAF mutants such as G466V and D594N harbor low to no kinase activity and function by binding tightly to RAS thus recruiting CRAF into hyperactivated heterodimers^15–17^. FDA-approved inhibitors such as vemurafenib have been successful in increasing progression-free survival of patients harboring hyperactive BRAF V600E mutations^18^. However, as with many kinase inhibitors, resistance occurs that renders patients insensitive to continued treatment^19^. Significant efforts have focused on creating drugs that target Class 2 BRAF mutations by inhibiting dimer formation, but adequate drugs have not yet been approved^10^. Therefore, there is a need for new and innovative therapies to address BRAF-driven cancers.

Proteolysis Targeting Chimeras (PROTACs) are heterobifunctional small molecules composed of a warhead that binds a protein of interest (POI), a flexible linker, and a ligand that binds an E3 ligase^20,21^. These molecules recruit an E3 ligase (e.g., VHL) to a POI to form a ternary complex. Upon complex formation, ubiquitin molecules are transferred to accessible lysines on the POI, marking it for proteasomal degradation. Importantly, by eliminating the entire protein scaffold, PROTACs are able to target both the enzymatic and non-enzymatic roles of disease-causing proteins. In recent years, our lab and others have made considerable progress in using PROTAC technology to induce degradation of proteins involved in disease, such as AR, ER, BRD4, RIPK2, BCR-Abl, EGFR, MET, p38 MAPK, BTK, and ERRα^22–32^. While traditional inhibitors require sustained target engagement for therapeutic effect, PROTACs simply require transient interaction, offering the ability to degrade proteins with limited target engagement^22^. Furthermore, the modular design of PROTACs allows for additional selectivity to be tuned into the small molecule, making it ideal for addressing difficult targets such as BRAF.

In this work, we use targeted protein degradation as a strategy to address mutant BRAF-driven cancer. We hypothesize that incorporating a BRAF inhibitor into PROTACs would allow for degradation of all BRAF isoforms. Despite the parent inhibitor, vemurafenib, being specific for Class 1 mutants only, we find that our PROTACs induce degradation of all three classes of BRAF mutants. Interestingly, BRAF^WT^ is degraded to a far lesser extent, and mechanistic studies show that BRAF^WT^ is spared due to a weaker ternary complex with the PROTAC and E3 ligase in cells. Furthermore, we are able to tune BRAF^WT^ degradation by increasing WT BRAF kinase activity. Overall, this study shows selective targeting of mutant BRAF with PROTAC technology and explores the mechanism that underlies the observed selectivity.

## Results

### SJF-0628 induces efficient and potent degradation of mutant BRAF but spares BRAF^WT^

**Fig. 1:**
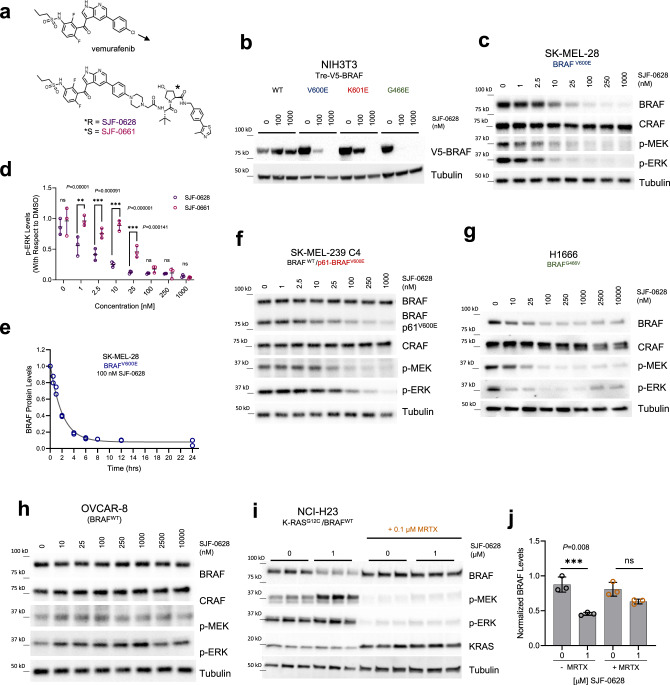
Vemurafenib-based PROTAC SJF-0628 induces degradation of mutant BRAF. **a** Chemical structure of vemurafenib and BRAF targeting PROTAC, SJF-0628, and its epimer, SJF-0661. SJF-0628 is composed of vemurafenib, a short piperazine-based linker, and a VHL recruiting ligand. SJF-0661 has an identical warhead and linker as SJF-0628 but contains an inverted hydroxyl group in the VHL ligand and is therefore unable to engage VHL to induce ubiquitination. **b** Inducible NIH3T3 cells expressing indicated V5-BRAF constructs (doxycycline 100–200 ng/mL, 24 h) treated with increasing amounts of SJF-0628. **c** SK-MEL-28 cells (homozygous BRAF^V600E^) treated with indicated amounts of SJF-0628 induced BRAF degradation and suppression of MEK and ERK phosphorylation. **d** Quantitation of ERK inhibition in SK-MEL-28 cells treated with SJF-0628 or SJF-0661 (mean ± SD, *n* = 3 biologically independent samples) *P* value calculated by multiple unpaired *t*-tests. **e** Quantitation of SJF-0628 treatment time course (100 nM) at indicated times in SK-MEL-28 cells shows maximal degradation within 4 h (*n* = 2 biologically independent samples). **f** SJF-0628 induces selective degradation of p61-BRAF^V600E^ mutant and inhibits MEK and ERK phosphorylation but spares BRAF^WT^ and CRAF in SK-MEL-239-C4 cells. **g** H1666 (heterozygous BRAF^G466V^) treated with SJF-0628 shows BRAF degradation, but incomplete suppression of ERK signaling. **h** BRAF^WT^ is spared by SJF-0628 in OVCAR-8 cells but induces slight activation of ERK phosphorylation. **i** Covalent inhibition of KRAS^G12C^ by MRTX849 in H23 cells hinders PROTAC induced BRAF^WT^ degradation (*n* = 3 biologically independent samples). **j** Quantification of **1i** (mean ± SD, *n* = 3 biologically independent samples). *P* value calculated by one-way ANOVA. Source data are provided as a Source Data file.

Although the utility of vemurafenib is limited to the treatment of tumors driven by BRAF^V600^ mutations, biochemical and binding studies show that these inhibitors also interact with BRAF^WT^, Class 2, and Class 3 BRAF mutants^11,15,18,33^. We therefore hypothesized that all BRAF isoforms would be susceptible to degradation by a vemurafenib-based PROTAC. Crystal structures of vemurafenib bound to BRAF^V600E^ reveal a solvent-exposed chloride at the para-position on the phenyl ring, which we posited would be ideal for linker addition (PDB: 3OG7)^34^ (Supplementary Fig. 1a,b). Pursuant to this, we iteratively optimized a lead vemurafenib-based PROTAC, SJF-0628, by coupling vemurafenib to a ligand for the von Hippel Lindau (VHL) E3 ligase using a rigid piperazine linker (Fig. 1a). In addition, we synthesized a degradation-incompetent control, SJF-0661, by inverting the stereocenter of the critical hydroxyl-proline group in the VHL ligand^22,35^. In NIH3T3 cells expressing doxycycline-inducible^14,15^, V5-tagged BRAF^WT^, Class 1, 2, or 3 BRAF mutations, SJF-0628 caused a dose-dependent decrease in the expression of all tested BRAF mutants, but spared BRAF^WT^, ARAF, and CRAF (Fig. 1b, Supplementary Fig. 1c). Mutant selectivity was also observed in 293 T-Rex cells expressing HA-tagged BRAF isoforms (Supplementary Fig. 1d).

To confirm these findings, we evaluated the ability of SJF-0628 to degrade endogenously expressed BRAF mutants in cancer cells. SJF-0628 treatment of SK-MEL-28 cells (homozygous BRAF^V600E^) resulted in a DC_50_ (half-maximal degradation) value of 6.8 nM and *D*_MAX_ (percent of maximal degradation) of >95% (Fig. 1c); similar results were seen in A375 cells (homozygous BRAF^V600E^) (Supplementary Fig. 2a). In SK-MEL-239 cells (heterozygous BRAF^V600E^), minimal BRAF degradation was observed, likely due to residual BRAF^WT^ although, there is a similarly sustained decrease in MAPK signaling (Supplementary Fig. 2b).

Treatment with 10 nM SJF-0628 caused maximal inhibition of MEK and ERK phosphorylation in SK-MEL-28 cells (Fig. 1c). As expected, while the epimer control, SJF-0661, did not decrease BRAF protein levels (Supplementary Fig. 2c), inhibition of MEK and ERK phosphorylation by this vemurafenib-based molecule was nonetheless observed. However, maximal suppression of p-ERK required 100 nM of SJF-0661, affirming a 10-fold increase in potency for the PROTAC from targeting both the enzymatic and non-enzymatic roles of BRAF (Fig. 1d). SJF-0628 induced near complete BRAF^V600E^ degradation within 4 h (Fig. 1e, Supplementary Fig. 2d), and BRAF^V600E^ degradation and p-ERK inhibition was sustained for up to 72 h (Supplementary Fig. 2e). A wash-out experiment of SJF-0628 after a 24 h treatment showed 30% recovery of BRAF levels and MAPK phosphorylation after 24 h, confirming the long acting and possibly catalytic effect of PROTACs (Supplementary Fig. 2f). BRAF^V600E^ degradation was prevented when cells were pre-treated with epoxomicin (proteasome inhibitor)^36^, MLN-4924 (neddylation inhibitor)^37^ or 100-fold excess vemurafenib, confirming SJF-0628 mediated protein loss is consistent with a PROTAC mechanism of action (Supplementary Fig. 2g). Treatment of VHL ligand alone did not affect MAPK phosphorylation, showing that the effect of the PROTAC is primarily due to degradation of BRAF (Supplementary Fig. 2h).

One clinically observed acquired resistance mechanism to vemurafenib is the aberrantly spliced BRAF mRNA transcript encoding an N-terminally truncated isoform that signals as a constitutive dimer (BRAF-p61^V600E^)^38^. In SK-MEL-239 C4 cells (BRAF^WT^/BRAF-p61^V600E^)^38^, SJF-0628 induced the degradation of the p61 dimer with a DC_50_ of 72 nM and *D*_MAX_ > 80%, while notably sparing BRAF^WT^ and CRAF (Fig. 1f). Similar results were seen in HCC-364 vr1cells (BRAF^WT^/BRAF-p61^V600E^)^39^ (DC_50_ of 147 nM, *D*_MAX_ > 90%) and 293 T-Rex cells overexpressing HA-BRAF-p61^V600E^ (Supplementary Fig. 3a, b). In SK-MEL-246 cancer cells^40^ (Class 2, BRAF^G469A^), SJF-0628 induced dose-dependent degradation of BRAF (DC_50_ = 15 nM, *D*_MAX_ > 95%) and concomitant inhibition of ERK phosphorylation while CRAF is slightly stabilized (Supplementary Fig. 3c).

Class 3 BRAF mutants are kinase dead or hypoactive and are frequently observed in NSCLC^15,41^. Unlike inhibitors, PROTACs offer a way to target the non-enzymatic/scaffolding role of these BRAF mutants by promoting their degradation. Treatment of NSCLC cell lines H1666 and CAL-12-T cells (Class 3, BRAF^G466V^; heterozygous and homozygous, respectively) with SJF-0628 caused a dose-dependent loss in BRAF protein levels in both cell lines (CAL-12T: DC_50_ = 23 nM, *D*_MAX_ > 90%) (H1666 cells: DC_50_ = 29 nM, *D*_MAX_ > 80%) (Fig. 1g, Supplementary Fig. 3d) as well as substantial p-ERK inhibition but showed slight stabilization at SJF-0628 concentrations higher than 1 µM.

### BRAF^WT^ activation via upstream effectors sensitizes it to SJF-0628 induced degradation at high concentrations

We next asked whether BRAF^WT^ is spared from SJF-0628-induced degradation in cancer cells as observed in the NIH3T3 overexpression system. In the ovarian carcinoma cell line OVCAR-8, SJF-0628 similarly induced minor BRAF^WT^ degradation and a slight induction of p-ERK (Fig. 1h). Given that activation shifts BRAF^WT^ from a closed (extended contact with N terminus) to an open conformation^42–44^, we sought to determine whether this conformational change can affect BRAF^WT^ susceptibility to SJF-0628 in cells with either amplified receptor tyrosine kinases (RTKs) or mutant RAS. In contrast to cells lacking constitutive upstream signaling, we observed ~30% degradation of BRAF^WT^ in A-431 cells (HER1 amplification) and ~50% degradation of BRAF^WT^ in SK-BR-3 cells (HER2 amplification) at PROTAC concentrations greater than 1 µM (Supplementary Fig. 4a, 8c-left panel). Despite the limited BRAF^WT^ degradation, we still observe paradoxical activation of MAPK signaling (increased p-ERK levels), likely due to PROTAC engagement of residual BRAF^WT^ and/or CRAF. Furthermore, addition of EGF to stimulate the MAPK pathway in OVCAR8 cells sensitized BRAF^WT^ to PROTAC-induced degradation (Supplementary Fig. 4b, c). In cells with mutant RAS (HCT-116, NCI-H23, and SK-MEL-30), the PROTAC also reduced BRAF^WT^ protein levels by 50–60% and caused ERK activation (Supplementary Fig. 4d–f). In H23 cells, reduction in BRAF expression was not accompanied by a change in its mRNA, suggesting that its degradation is induced by SJF-0628 (Supplementary Fig. 4g).

Our results suggest that the activated conformation of BRAF may be sensitized to PROTAC-induced degradation. Accordingly, we tested whether inhibition of upstream signaling in these cells, which would reduce BRAF activation, would also reduce the effects of the PROTAC. In SK-BR-3 cells, the HER2/EGFR kinase inhibitor lapatinib (2-hour pre-treatment) reduced SJF-0628-dependent BRAF^WT^ degradation from 48% to 10% (Supplementary Fig. 4h, i). Similarly, in NCI-H23 cells, pre-treatment with the K-RAS^G12C^ inhibitor, MRTX849, for 2 h also desensitized BRAF^WT^ to the PROTAC: from 50% BRAF^WT^ degradation in cells treated with the PROTAC alone to 20% degradation in the MRTX849 pre-treated cells (Fig. 1i, j). Thus, sensitivity of BRAF^WT^ to SJF-0628-mediated degradation is associated with activation of its upstream effectors.

### Exploration of SJF-0628 mutant selectivity shows BRAF^WT^ is unable to form a stable ternary complex in cellulo

The selectivity of SJF-0628 for mutant BRAF over BRAF^WT^ suggests that it may have little on-target toxicity and therefore a wide therapeutic index in patients. However, the mechanism of this selectivity is not clear since vemurafenib binds BRAF^WT^ as well as BRAF mutants. During lead optimization, several vemurafenib-based PROTACs were synthesized with varied linker lengths and composition. Similar to SJF-0628, these PROTACs selectively induced degradation of mutant BRAF (Supplementary Fig. 5a, b). Furthermore, during the preparation of this manuscript, two groups published cereblon-based PROTACs, which incorporated vemurafenib or BI882370, that induces BRAF^V600E^ degradation and also spared BRAF^WT^ ^45,46^. As this phenomenon appears to hold true for multiple BRAF-targeting PROTACs, we explored the mechanism that underlies the observed selectivity.

**Fig. 2:**
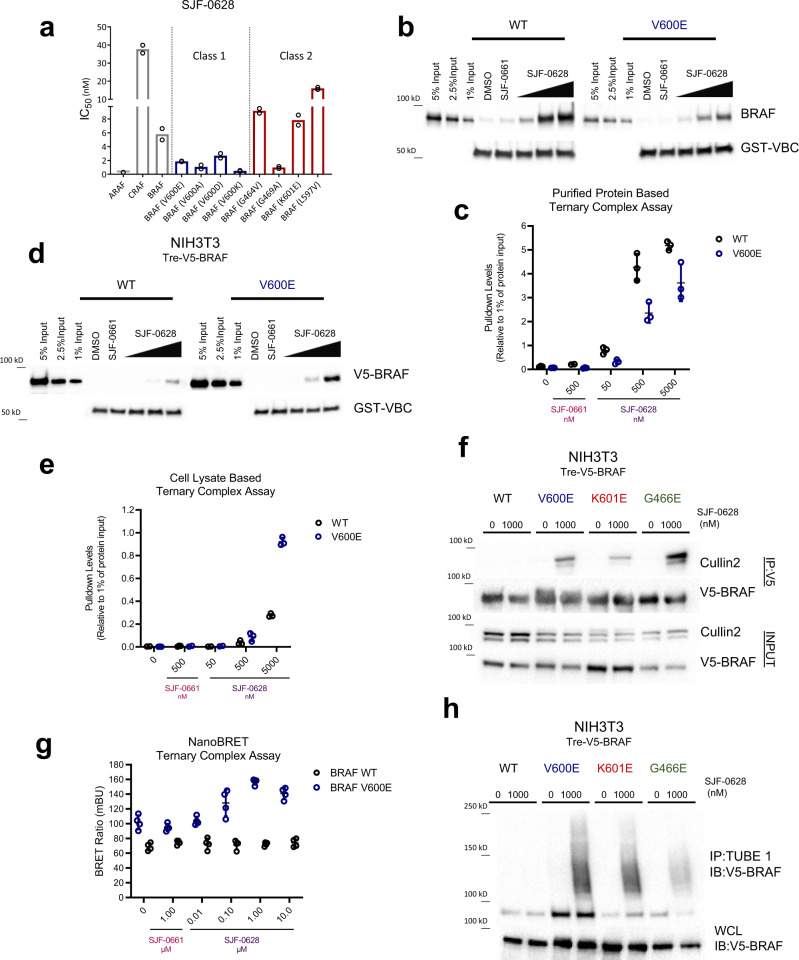
BRAF^WT^ is unable to form a PROTAC-induced ternary complex in cells and thus not degraded. **a** IC_50_ values of radiolabeled kinase assay for WT RAF and Class 1 and 2 BRAF mutants (mean, *n* = 2 biologically independent experiments). Plotted values shown in Table 1. **b** Purified protein ternary complex assay. GST-VBC (VHL, Elongin B, Elongin C) is immobilized on glutathione beads and incubated with DMSO, SJF-0661 (500 nM) or increasing concentrations of SJF-0628 and purified full length-BRAF to observe VBC:PROTAC:BRAF ternary complex. **c** Quantification of **2b** with respect to 1% input (mean ± SD, *n* = 3 biologically independent samples). Replicates shown in source data; WT = black circles, V600E = blue circles. **d** Cell lysate based ternary complex assay (as described in **b**) but using NIH3T3 cell lysates (doxycycline 800 ng/mL) containing V5-BRAF^WT^ or V5-BRAF^V600E^ as input. **e** Quantification of **2d** with respect to 1% input (mean ± SD, *n* = 3 biologically independent samples). Replicates shown in source data. WT = black circles, V600E = blue circles. **f** NIH3T3 cells expressing indicated V5-BRAF treated with DMSO or 1 µM SJF-0628 for 1-hour followed by immunoprecipitation of V5-BRAF. **g** NanoBRET ternary complex assay. HEK293T cells ectopically expressing NanoLuc-BRAF(donor) and HaloTag-VHL covalently labeled with a HaloTag 618 ligand (acceptor) were treated with DMSO, epimer SJF-0661(1 μM) or indicated concentration of SJF-0628 for 3 h. Data represented as BRET ratio (mean ± SD, *n* = 4 biologically independent experiments); WT = black circles, V600E = blue circles. **h** Tandem Ubiquitin Binding Entities 1 (TUBE1) pull down of tetra-ubiquitinated proteins in NIH3T3 cells expressing indicated V5-BRAF after 1-hour treatment with vehicle or SJF-0628. Immunoblotted for V5-BRAF. Source data are provided as a Source Data file.

We evaluated the distinct mechanistic steps that PROTACs undertake to induce degradation: target engagement, ternary complex formation (target protein: PROTAC: E3 ligase) and target ubiquitination. In a radioactive in vitro assay of purified RAF kinase activity, SJF-0628 potently inhibited both BRAF^WT^ (IC_50_ = 5.8 nM) and BRAF^V600E^ (IC_50_ = 1.87 nM). (Fig. 2a; Table 1) Generally, Class 2 mutants bound SJF-0628 with weaker affinity, but nevertheless are successfully degraded in cells; Class 3 mutants were not tested due to their inherent weak kinase activity. Furthermore, SJF-0628 induces paradoxical activation of MAPK signaling, showing that BRAF^WT^ engagement is also achieved in cells. Thus, binary binding is not the basis of isoform degradation selectivity by SJF-0628.

**Table 1:** Table of IC_50_ values from radio labeled kinase assay (*n* = 2 biologically independent experiments).

Kinase:	SJF-0628 IC_50_ (nM)
ARAF	0.27
CRAF	37.6
BRAF	5.80
BRAF (V600E)	1.87
BRAF (V600A)	1.06
BRAF (V600D)	2.68
BRAF (V600K)	0.49
BRAF (G464V)	9.18
BRAF (G469A)	0.98
BRAF (K601E)	7.84
BRAF (L597V)	16.0

To determine whether differences in ternary complex formation explain the differential degradation observed, we performed a pulldown experiment by immobilizing recombinant GST-tagged VHL/Elongin B/Elongin C (VBC) on glutathione-sepharose and incubating the beads with purified full-length BRAF and increasing amounts of PROTAC, with the goal of detecting a VHL: PROTAC: BRAF trimer. Indeed, there was a dose-dependent increase of BRAF^WT^ and BRAF^V600E^ complexed with the VBC at comparable levels (Fig. 2b, c). In fact, BRAF^WT^ appeared to form a stronger ternary complex than BRAF^V600E^. Hence the innate capacity to form a trimer therefore does not contribute to BRAF isoform selectivity of degradation. However, mutant and BRAF^WT^ adopt different conformations and form unique complexes within cells^4,47^. Thus, we hypothesized that BRAF cellular conformations or associated proteins may affect trimer formation. To investigate this, we performed pull-down assays using NIH3T3 cell lysates expressing V5-BRAF^WT^ or V5-BRAF^V600E^. Interestingly, we found that while BRAF^WT^ forms a ternary complex with VHL in this lysate-based assay, this occurs to a lesser extent than it does for BRAF^V600E^ (~3 fold greater than BRAF^WT^ at 5000 nM), as well as for BRAF^K601E^ and BRAF^G466E^ (Fig. 2d, e and Supplementary Fig. 6a). This result suggested that the in vitro trimer formation system using purified recombinant BRAF may not fully recapitulate PROTAC-induced complex formation in cells.

Therefore, we sought to further examine ternary complex formation in cells. We treated NIH3T3 cells with SJF-0628 for 1 h (to minimize degradation) and pulled down V5-BRAF and any associated VHL E3 ligase components. While SJF-0628 induced an interaction of all three mutant BRAF classes with Cullin 2, the E3 adaptor of VHL, it did not promote BRAF^WT^ trimer formation (Fig. 2f). At higher concentrations, we observe some ternary complex formation with BRAF^WT^, but much less than that seen with mutant BRAF (Supplementary Fig. 6b, c). To further examine PROTAC induced proximity in intact cells, we employed the NanoLuc bioluminescent resonance energy transfer (NanoBRET) system comprised of transiently co-expressed C-terminal tagged NanoLuc–BRAF (donor) isoforms and HaloTAG-VHL labeled with a HaloTAG NanoBRET 618 ligand (acceptor). In this system, while SJF-0628 caused an increase in BRET signal in cells expressing BRAF^V600E^, there was no observed increase in cells expressing BRAF^WT^ at concentrations as high as 10 μM (Fig. 2g). We did not observe an increase in BRET ratio in the absence of BRAF or VHL, confirming that the BRET signal observed is indeed due to ternary complex formation (Supplementary Fig. 6d). Furthermore, while all three mutant classes are ubiquitinated in cells, BRAF^WT^ is not (Fig. 2h, Supplementary Fig. 6e, f). Overall, these studies show that BRAF^WT^ weakly associates with the E3 ligase complex in the cellular milieu, and this leads to minimal ubiquitination and degradation as compared to mutant BRAF; hence, the mutant selectivity of the PROTAC.

### Relief of negative feedback sensitizes BRAF^WT^ to SJF-0628 induced degradation

Our results suggest that BRAF^WT^ conformation and complex heavily influences its degradability. Therefore, we directly interrogated how these properties affect the ability to induce BRAF^WT^ degradation. Studies have shown that MEK inhibition potentiates the activated state of RAF by attenuating feedback inhibition from downstream effectors to stabilize/increase RAF dimerization and association with RAS^48,49^. Therefore, we hypothesized that MEK inhibition would allow for enhanced BRAF^WT^ degradation.

**Fig. 3:**
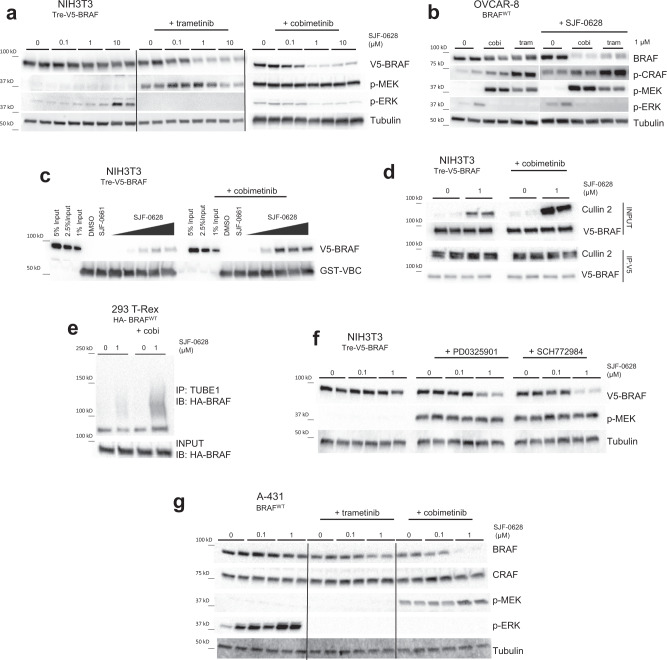
BRAF-activating MEK inhibitors also sensitize BRAF to PROTAC-induced ubiquitination and degradation. **a** NIH3T3 cells with trametinib (1 µM, 5 h) or cobimetinib (500 nM, 3 h) pre-treatment subsequently treated with increasing amounts of SJF-0628 (20 h) promote degradation of BRAF^WT^ and show a marked increase in p-MEK (*n* = 2 biologically independent samples). **b** OVCAR8 cells pre-treated with cobimetinib and trametinib (1 µM, 2 h) promote MEK and CRAF phosphorylation as well as BRAF degradation in the presence of SJF-0628 (*n* = 2 biologically independent samples). **c** Cell lysate-based ternary complex assay shown in 2c but using NIH3T3 lysates expressing BRAF^WT^ and pre-treated with DMSO or 1 µM cobimetinib for 3 h. Cobimetinib pre-treatment promotes ternary complex formation. **d** V5-BRAF immunoprecipitation in NIH3T3 cells pre-treated with 1 µM of cobimetinib (2 h) followed by treatment of SJF-0628 for 2.5 h. **e** TUBE1 pulldown in 293 T-Rex cells stably expressing HA- BRAF^WT^ treated with cobimetinib (cobi) (2 h, 1 µM) and subsequently treated with SJF-0628 (2 h). **f** NIH3T3 cells pre-treated with 1 µM PD0325901 (MEK inhibitor) or SCH772984 (ERK inhibitor) for 3 h followed by treatment with indicated amount of SJF-0628 for 20 h. **g** A431 cells pre-treated with GDC-0623 and cobimetinib (500 nM for 3 h) then treated with SJF-0628 for 20 h. Source data are provided as a Source Data file.

To test this, we pre-treated NIH3T3 cells with the allosteric MEK inhibitor, trametinib, followed by increasing doses of SJF-0628. Interestingly, trametinib addition caused dose-dependent PROTAC-induced BRAF^WT^ degradation, as well as increased MEK phosphorylation (Fig. 3a, Supplementary Fig. 7a); trametinib effectiveness was confirmed by the minimal ERK phosphorylation observed. Trametinib pre-treatment did not prevent BRAF^V600E^ degradation, nor did it promote epimer-induced degradation of BRAF^V600E^ or BRAF^WT^ in NIH3T3 cells (Supplementary Fig. 7b, c). SJF-0628 also induced BRAF^WT^ degradation in NIH3T3 cells that were pre-treated with cobimetinib – a second, structurally distinct allosteric MEK inhibitor (Fig. 3a). In OVCAR-8 cells, pre-treatment with cobimetinib or trametinib also enabled PROTAC-induced BRAF^WT^ degradation while increasing MEK and CRAF phosphorylation (Fig. 3b). Cobimetinib pre-treatment stimulated MEK phosphorylation within 30 min, and enabled PROTAC-induced degradation of BRAF^WT^ within 4 h, with complete degradation observed after 12 h (Supplementary Fig. 7d). No changes in BRAF^WT^ mRNA levels were observed (Supplementary Fig. 7e) confirming that BRAF^WT^ downregulation by SJF-0628 in the presence of cobimetinib is, indeed, post-translational. Moreover, we observed markedly increased trimer formation in cell lysates pre-treated with cobimetinib (Fig. 3c). In addition, MEK inhibitor-pretreated cells generated a 4-fold increase in PROTAC-induced Cullin-2 association with BRAF^WT^ (Fig. 3d) and increased SJF-0628-dependent BRAF^WT^ ubiquitination (Fig. 3e) showing that the BRAF^WT^ degradation observed occurred via a PROTAC mechanism of action. These data, taken together, support our hypothesis that the activated conformation drives the ability of the PROTAC to degrade BRAF^WT^.

To rule out other aspects of MEK inhibition that may promote BRAF^WT^ degradation by SJF-0628, we undertook a series of pharmacological studies. In addition to inhibiting negative feedback and increasing BRAF activity, cobimetinib and trametinib have also been shown to decrease BRAF^WT^ association with MEK^50^. As such, MEK might hinder BRAF^WT^ ternary complex formation with SJF-0628 and VHL, preventing degradation of BRAF^WT^. To investigate this, we pre-treated cells with an early generation MEK inhibitor, PD0325901, known to stabilize the BRAF:MEK complex^51^ (Supplementary Fig. 8a). However, cells pretreated with PD0325901 also enabled PROTAC-mediated BRAF^WT^ degradation (~80% degradation at 1 μM) (Fig. 3f). We hypothesized that if relief of MAPK negative feedback promotes BRAF^WT^ degradation, ERK inhibition would do the same. As predicted, pretreatment with the selective ERK inhibitor SCH772984^52^ at 1 µM enabled ~90% degradation of BRAF^WT^ by the PROTAC (Fig. 3f). SCH772984 also does not disrupt BRAF-MEK association, further demonstrating that the presence of MEK does not affect BRAF^WT^ degradation (Supplementary Fig. 8a).

GDC-0623 is an allosteric MEK inhibitor which binds MEK in a manner that sequesters BRAF and hinders dimerization with itself or CRAF (Supplementary Fig. 8a) and membrane localization^50,53^. Therefore, treatment with GDC-0623 dampens relief of feedback induced signaling on RAF kinase activity. We hypothesized that if the BRAF^WT^ conformation induced by the previously tested MEK/ERK inhibitors is primarily responsible for enabling the kinase’s degradation by the PROTAC, then GDC-0623 would enable significantly less degradation. As expected, GDC-0623 pre-treatment permitted only minimal PROTAC-dependent degradation of BRAF^WT^ – far less than was seen in parallel treatment with cobimetinib (Fig. 3g, Supplementary Fig. 8b, c). These results further show that SJF-0628 selectively induces degradation of BRAF^WT^ in its active conformation. Indeed, studies such as Rock et al. show that all three mutant BRAF classes (including kinase dead mutations) favor an open, active conformation^54^. Thus, by stimulating BRAF^WT^ activity (e.g., RTK upregulation, RAS mutations, relief of negative feedback), we promote an open conformation which is susceptible to increased ternary complex formation and therefore degradation.

### SJF-0628 successfully inhibits cell growth in mutant-BRAF driven cancer cells

**Fig. 4:**
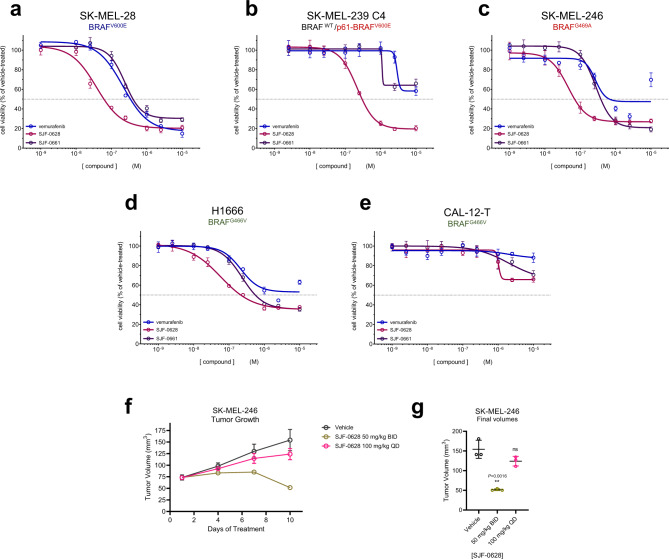
SJF-0628 outperforms vemurafenib in inhibiting growth of cell lines expressing mutant BRAF. **a** Cell proliferation assay in SK-MEL-28 cells treated with increasing amounts of vemurafenib, SJF-0628, or SJF-0661 for 3 days (mean ± SD, *n* = 3 biologically independent samples). EC_50_ = 215 ± 1.09 nM, 37 ± 1.2 nM, and 243 ± 1.09 nM, respectively; vemurafenib = blue, SJF-0628 = burgundy, SJF-0661 = purple. **b** Cell proliferation assay in vemurafenib resistant SK-MEL-239-C4 cells treated with increasing amounts vemurafenib, SJF-0628, or SJF-0661 for 5 days (mean ± SD, *n* = 3 biologically independent samples); vemurafenib = blue, SJF-0628 = burgundy, SJF-0661 = purple. **c** Cell proliferation assay in SK-MEL-246 (Class 2) cells treated with increasing amounts vemurafenib, SJF-0628, or SJF-0661 for 5 days (mean ± SD, *n* = 3 biologically independent samples); vemurafenib = blue, SJF-0628 = burgundy, SJF-0661 = purple. **d** SJF-0628 EC50 = 218 nM ± 1.06 c,H1666 cells treated with SJF-0628, vemurafenib, or SJF-0661 for 5 days (mean ± SD, *n* = 3 biologically independent samples); vemurafenib = blue, SJF-0628 = burgundy, SJF-0661 = purple. **e** Treatment of CAL-12-T cells with vemurafenib, SJF-0628, or SJF-0661 for 5 days shows minimal effect on cell viability (mean ± SD, *n* = 3 biologically independent samples). **f** Results of an efficacy study in SK-MEL-246 tumor xenografts implanted in female athymic mice showing tumor regression with 50 mg/kg IP twice daily (mean ± SD, *n* = 3 biologically independent animals). **g** Scatter plot result of final tumor volumes of SKMEL-246 xenografts treated with SK-MEL-246 (mean ± SD, *n* = 3 biologically independent animals). *P* value calculated by unpaired *t*-test. Source data are provided as a Source Data file.

Next, we compared the effects on cell growth of inhibiting both the enzymatic and scaffolding roles of BRAF using SJF-0628, with those of an ATP-competitive inhibitor that targets only its catalytic function (vemurafenib and degradation-incompetent epimer, SJF-0661). In SK-MEL-28 cells (Class 1, BRAF^V600E^), vemurafenib and SJF-0661 inhibited cell growth with an EC_50_ of 215 ± 1.09 nM and 243 ± 1.09 nM, respectively, while SJF-0628 showed an EC_50_ of 37 ± 1.2 nM (Fig. 4a). The 6-fold increase in potency of SJF-0628 occurred despite the compounds having similar in vitro binding (vemurafenib = 27 nM, SJF-0628 = 39 nM, SJF-0661 = 64 nM) (Supplementary Fig. 9a, Supplementary Table 2). In SK-MEL-239 C4 cells (BRAF^WT^/BRAF-p61^V600E^), while vemurafenib and SJF-0661 had a minimal effect, SJF-0628 induced ~80% decrease in cell growth with an EC_50_ of 218 nM ± 1.06 (Fig. 4b). This result shows that targeted degradation can be used to overcome acquired resistance to BRAF inhibitor-based therapies. A 5-day treatment in SK-MEL-246 cells (Class 2, BRAF^G469A^) SJF-0628 efficaciously inhibited cell growth with and EC_50_ of 45 ± 1.11 nM and the epimer showed and an EC_50_ 278 ± 1.07 nM. However, vemurafenib caused some inhibition of SK-MEL-246 cellular growth at concentrations above 1 μM which was not sustained at 10 μM (Fig. 4c). In H1666 cells (Class 3, BRAF^G466V^), SJF-0628 was able to induce 65% cell growth inhibition while vemurafenib showed less than 50% (Fig. 4d). Despite causing >70% inhibition of p-ERK (Supplementary Fig. 3d), SJF-0628 showed minimal inhibition of cell growth in CAL-12-T cells (Class 3, BRAF^G466V^) (Fig. 4e).

SJF-0628 causes potent degradation of all BRAF mutant classes and has minor effects on WT RAF. This result suggests that the PROTAC will be effective in treating tumors driven by these mutations with minimal on target toxicity, thus we tested the effects of SJF-0628 in an A375 (BRAF^V600E^) murine xenograft model. Mice treated with SJF-0628 (three days; 50 mg/kg or 150 mg/kg) showed marked degradation of BRAF in the xenograft at both concentrations (*D*_MAX_ > 90%) (Supplementary Fig. 9b). Because SJF-0628 successfully induced degradation in vivo, we tested its effect on tumor growth in the SK-MEL-246 melanoma xenograft model (Class 2, BRAF^G469A^). Strikingly, while once daily 100 mg/kg treatment showed a minor response, twice daily treatment of 50 mg/kg induced tumor shrinkage beyond the initial tumor size within 10 days (Fig. 4f, g). We did not observe a significant body weight loss with either dose (Fig. S9C). Thus, through targeted degradation, SJF-0628 is successfully able to exhibit a significant antitumor effect.

## Discussion

Each BRAF mutation alters how the protein signals in distinct ways, therefore, careful consideration must be taken to select the appropriate inhibitor. Despite tremendous effort to create therapies that target diverse BRAF mutations, all three currently FDA-approved drugs target Class 1 BRAF mutants alone. Furthermore, resistance to current drugs inevitably occurs. Thus, we have developed a vemurafenib-based PROTAC, SJF-0628, that outperforms vemurafenib in inhibiting MAPK signaling and growth of BRAF^V600E^-driven cancer cells. Importantly, the mutant BRAF-targeting PROTACs described here mostly spare WT RAF, thus widening the potential therapeutic window of this new class of anti-tumor drugs.

We find that measuring ternary complexes (BRAF:PROTAC:VHL) in cells or cell lysates to be more predictive of degradation than in vitro studies with purified proteins. Interestingly, similar results were observed by Posternak et al. with cereblon recruiting BRAF-targeting PROTAC^45^. Our in vitro pull-down assays likely contained highly dimerized BRAF in the active conformation, resulting in artificially high levels of ternary complex formation. However, in cells BRAF exists in a closed inactive conformation, which is less conducive to ternary complex formation allowing it to escape degradation. So by promoting its activation, BRAF^WT^ adopts an open conformation, similar to mutant BRAF, and is thus susceptible to SJF-0628-induced degradation. This finding suggests that intracellular protein conformation can affect PROTAC-induced degradation. As PROTACs require induced protein–protein interactions to function, it is important to consider protein interactions within the cellular context that might encourage or discourage degradation. Importantly for the development of new PROTACs, we show that we can control induced protein degradation (i.e., BRAF^WT^) without modifying the PROTAC itself but by manipulating its signaling pathway.

While preparing this manuscript, two groups described cereblon-based PROTACs that degrade BRAF^V600E^ but spares BRAF^WT^ ^45,46^. Beyond this, we also successfully target vemurafenib-resistant BRAF mutations. This includes mutants that have both acquired (p61 V600E) and intrinsic (Class 2) resistance to vemurafenib. Furthermore, we show that SJF-0628 can be used to successfully target Class 2 mutants in vivo. In addition, we make Class 3 BRAF mutants, which cannot be targeted with traditional small molecule inhibitors, therapeutically accessible through targeted degradation. Indeed, this is another early demonstration of PROTAC induced degradation of a pseudokinase. Thus, using PROTACs, we are able to expand the druggable space to a class of proteins with immense cancer relevance (HER3, ROR2, etc.). In summary, this study demonstrates that the PROTAC technology is an attractive strategy for targeting difficult oncoproteins such as mutant BRAF.

## Methods

### PROTAC treatment and immunoblotting

Cells were plated in 6 well dishes (5 × 10^5^–8 × 10^5^ cells) and allowed to attach overnight. Cells were treated with SJF-0628 or SJF-0661 for 20–24 h (unless otherwise stated). The plates were then placed on ice and washed 1x with chilled PBS and lysed in buffer containing 25 mM Tris-HCl [pH 7.4], 0.25% sodium deoxycholate, 150 mM NaCl, 1% Triton X-100, supplemented with protease inhibitors (1x Roche protease inhibitor cocktail) and phosphatase inhibitors (10 mM NaF, 1 mM Na_3_OV_4_, and 20 mM β-glycerophosphate). Lysates were then cleared at 21,000 *g* for 10 min at 4 °C. Protein concentrations of the supernatants were then quantified using a Pierce BCA Protein Assay. 12–40 µg of protein were separated using a gradient (4–20%) Criterion TGX precast gel and transferred unto a nitrocellulose membrane. The membranes were then blocked in 5% non-fat milk in TBST (Tris-buffered Saline with Tween 20) for 1 h before probing with the indicated primary antibody overnight. Membranes were imaged using Bio-Rad Image Lab software using ECL prime detection reagent (GE Healthcare, RPN2232 or ThermoScientific, 34095).

### Cell proliferation

Cells (25,000 to 5000) were seeded in 96 well plates and treated with compound for the indicated lengths of time (between 72 and 96 h). 2 mg/ml MTS (Promega Corp., Madison, WI: G5421) and 25 μM phenazine methosulfate (Sigma, St. Louis, MO) were combined 19:1 and then added to cells (1 volume combined reagent: 5 volumes medium) and incubated for 1–3 h. Mitochondrial reduction of MTS to the formazan derivative was monitored by measuring the medium’s absorbance at 480 nm using a Perkin Elmer Envision Plate reader.

### Protein purification

For the expression of GST-tagged VHL:Elongin B:Elongin C (herein referred to as GST-VBC), wild-type human VHL, Elongin B, and Elongin C were co-expressed in *E. coli*. BL21(DE3) cells were co-transformed with pBB75-Elongin C and pGEX4T-2-VHL-rbs-Elongin B and selected in LB medium containing carbenicillin (100 µg mL^−1^) and kanamycin (25 µg mL^−1^) at 37 °C until OD 600 = 0.8, at which point the culture was chilled to 16 °C and induced with 0.4 mM IPTG for 16 h. Cells were homogenized and lysed using a Branson digital sonifier with lysis buffer composed of 50 mM Tris [pH 8.0], 200 mM NaCl, 5% glycerol, 5 mM DTT containing a 1 X protease inhibitor cocktail tablet (Roche). Clarified cell lysate was applied to glutathione sepharose 4B beads (GE Life Science) and gently rotated for 2 h at 4 °C. Beads were washed with four column volumes of lysis buffer, followed by four column volumes of elution buffer (50 mM Tris pH 8.0, 200 mM NaCl, 10 mM glutathione). Eluted protein was assessed for identity and purity via Coomassie staining of sample run on an SDS-PAGE gel and pure elutions were pooled, concentrated, and diluted in ion-exchange buffer A (30 mM Tris pH 8.0, 5% glycerol, 1 mM DTT) until the salt concentration was 50 mM, before loading onto a Mono Q 5/50 GL column (GE Life Sciences). The protein was subjected to a linear gradient of NaCl (0–500 mM NaCl) using ion-exchange buffer B (30 mM Tris 8.0, 1 M NaCl, 5% glycerol, 1 mM DTT). Fractions were then assessed for purity via Coomassie, pooled, concentrated, and run on a Superdex-200 column (GE Life Sciences) using size-exclusion buffer (30 mM Tris pH 8.0, 100 mM NaCl, 10% glycerol, 1 mM DTT). Pure fractions of GST-VHL were pooled, concentrated to ~5 mg mL^−1^, aliquoted, and flash-frozen before storing at −80 °C.

### Ternary complex assays

Glutathione sepharose 4B was washed twice with water and then blocked for 1 h at room temperature with 10% BSA in TBST. The beads were then washed again twice with TBST and once with wash buffer (50 mM HEPES pH 7.5, 150 mM NaCl, 1 mM DTT, 0.01% NP40, 5 mM MgCl_2_, 10% glycerol) and then purified GST-VBC was immobilized for 2 h at 4 °C at 2.5 pmole per μL of beads. The beads were then washed thrice with wash buffer, resuspended and BRAF WT or V600E protein was added at 500 nM per 50 μL reaction with 5 μL of beads. The bead:BRAF mixture was then aliquoted to separate tubes and PROTAC was added at the indicated concentration (PROTACs were intermediately diluted in 50% DMSO) and this was incubated at 4 °C for 2 h. The beads were washed 4 times with 1 mL column of TBST and then eluted with SDS loading buffer.

For experiments in which the input substrate is a whole cell lysate, the sample was prepared as follows: 15 mm dishes of confluent NIH3T3 cells were doxycycline induced overnight, after which the cells were washed with DPBS and lysed using lysis buffer (50 mM Tris-HCl, pH 7.4, 015 M NaCl, 1 mM EDTA,1% NP40, 10% glycerol). The lysate was cleared by centrifugation and then added to the beads as an input substrate, as above. For MEK inhibitor comparison, NIHT3 cells were pre-treated with 1 µM of cobimetinib for 3 h.

### NanoBRET ternary complex assay

Full-length BRAF (WT and V600E) was cloned into the pNLF1-C [CMV/Hygro] vector. HEK 293 T cells were seeded into 6 well dishes (~75% confluent) and co-transfected with HaloTag-VHL (Promega; N273A) and C-terminal nanoLUC-tagged BRAF. Transfection was performed using a ratio of 1:2 DNA to Lipofectamine 2000. After 24 h, 1 × 10^4^ cells were seeded into white 96 well plates (Costar Cat# 3610) in Opti-MEM™ I Reduced Serum Medium supplemented with 4% FBS and 0.1 mM HaloTag NanoBRET 618 ligand or DMSO. The next day, the cells were pre-treated with 2 μM MG-132 for 1 h then treated with indicated amount of drug for 3 h. 6X NanoBRET Nano-Glo Substrate in Opti-MEM was then added to each well and the plate was read using a Perkin Elmer Envision. Dual filtered luminescence was measured with a 430 nm (donor, HaloTag NanoBRET ligand) and a 615 nm filter (acceptor, HaloTag NanoBRET ligand). Background corrected (cells without HaloTag 618 ligand) nanoBRET ratios were calculated to determine intracellular ternary complex formation.

### Cellular immunoprecipitation and ubiquitination assay

Doxycycline-induced NIH3T3 cells or 293 T-Rex cells that express indicated BRAF isoform were seeded in 10 cm dishes overnight. Cells were then treated for 1 h with PROTAC or DMSO. Cells were then placed on ice, washed with ice-cold 1X PBS, and lysed in 500 µL modified 1X lysis buffer (50 mM Tris-HCl, pH 7.4, 0.15 M NaCl, 1 mM EDTA, 1% NP40, 10% glycerol) containing 5 mM 1,10-phenanthroline monohydrate, 10 mM N-ethylmaleimide, 20 µM PR-619, and 1X protease inhibitor cocktail (Roche). Lysates were spun down at 14,000 × *g* at 4 °C for 10 min. Equal amounts of lysate was aliquoted onto 20 µL (bed volume) of anti-V5-beads (Sigma, A7345). V5-containing proteins were immunoprecipitated from lysates for 2 h at 4 °C with gentle rotation, after which samples were spun down at 6000 × *g* at 4 °C for 2 min and the beads were washed 4 times with DPBS. Beads were resuspended in 1X lithium dodecyl sulfate (LDS) sample buffer containing 5% 2-mercaptoethanol (ß-ME). Immunoprecipitated protein was eluted off the beads by heating at 95 °C for 5 min and the supernatant was run on an SDS-PAGE gel and evaluated for the presence of immunoprecipitated V5-tagged proteins, as well as Cullin 2. Input refers to the normalized input lysate loaded onto V5-sepharose beads.

TUBE1 immunoprecipitation experiments were carried out exactly as described above, except that equal amount of lysate was loaded onto 20 µL TUBE1 agarose (LifeSensors) resin per sample and washed with TBST.

### Radiolabeled kinase assays

Kinase assays were performed by Reaction Biology Corps by their protocol in duplicate using K_m_ amounts of ATP calculated for each kinase.

### Elisa kinase inhibition assay

Kinase assays were performed by Carna Biosciences by their protocol in duplicate using K_m_ amounts of ATP calculated for each kinase.

### RT-PCR

Cells were seeded in 12 well plates and treated as described. RNA was isolated with the RNeasy Mini Kit (QIAGEN) and 1 μg of total RNA was reverse transcribed using the High Capacity cDNA Reverse Transcription Kit (Applied Biosystems). SYBR Green PCR master mix (Kapa Biosystems) was used for qRT-PCR samples were performed and analyzed in triplicate. Relative RNA expression levels were calculated using the ddCt method and normalized to control samples and beta-tubulin was used for normalization. Primers used in this study are included in Supplementary Table 1.

### A375 xenograft study

5 million A375 cells were subcutaneously implanted in female nu/nu mice. Tumors were randomized after a period of 10 days into groups with an average tumor size of 350 mm^3^, and treated with vehicle (5% DMSO, 5% EtOH, and 20% Solutol HS15 in D5W), 50 mg/kg SJF-0628, or 150 mg/kg SJF-0628 (4 mice per arm) intraperitoneally once a day for 3 days. Mice were sacrificed 8 h after the final dose. Tissues were harvested, flash frozen, and lysed in 1X cell lysis buffer (Cell Signaling #9803) supplemented with protease and phosphatase inhibitors. Harvested tumors were disrupted using metal beads in a Tissuelyser. Homogenates were normalized for protein content and analyzed using SDS-PAGE and Western blotting. All studies were performed in compliance with institutional guidelines under an IACUC approved protocol.

### SK-MEL-246 xenograft study

10 million SK-MEL-246 cells were subcutaneously implanted in female nu/nu mice. The tumor volumes and mice weights were measured twice a week after the implantation. The i.p. treatments with vehicle (5% DMSO, 5% EtOH, and 20% Solutol HS15 in D5W), 50 mg/kg (BID) or 100 mg/kg SJF-0628 (QD), (3 mice/group) were started when the tumor volumes reached an average of 100 mm^3^. All studies were performed in compliance with institutional guidelines under an Institutional Animal Care and Use Committee (IACUC) approved protocol. Investigators were not blinded when assessing the outcome of the in vivo experiments.

### Quantitation and statistical analysis of Western blots

Western blot data was quantified by using the band feature in Image Lab, and values were averaged and analyzed in GraphPad Prism. DC_50_ and *D*_MAX_ values were fitted using a three parameter [inhibitor] versus response and reported directly from the Prism output. Mean ± SD and unpaired *t*-tests were performed in GraphPad Prism.

### Structure visualization

Atomic resolution structures were visualized using Molecular Operating Environment software (MOE-2019.0102).

### Statistics and reproducibility

All experiments were performed 2 or more independent times with similar results.

### Reporting summary

Further information on research design is available in the Nature Research Reporting Summary linked to this article.

## Supplementary information

Reporting Summary

Supplementary Information

## Data Availability

Linker positioning was determined using structure of vemurafenib bound to BRAF (PDB:3OG7). Source data are provided with this paper. All other data are available from the corresponding author on reasonable request. Source data are provided with this paper.

## Source data

Source Data
