# Supplementary material for: Mutant-selective degradation by BRAF-targeting PROTACs
Source: Nat Commun. 2021 Feb 10;12:920. doi: 10.1038/s41467-021-21159-7 (PMC7876048; doi:10.1038/s41467-021-21159-7)
Supplement: Supplementary file 1 — Reporting Summary [file 41467_2021_21159_MOESM1_ESM.pdf]

nature research

Corresponding author(s): Craig Crews
Last updated by author(s): 1-7-2021

Reporting Summary

Nature Research wishes to improve the reproducibility of the work that we publish. This form provides structure for consistency and transparency in reporting. For further information on Nature Research policies, see our Editorial Policies and the Editorial Policy Checklist.

Statistics

For all statistical analyses, confirm that the following items are present in the figure legend, table legend, main text, or Methods section.

n/a	Confirmed	
☐	☒	The exact sample size (*n*) for each experimental group/condition, given as a discrete number and unit of measurement
☐	☒	A statement on whether measurements were taken from distinct samples or whether the same sample was measured repeatedly
☐	☒	The statistical test(s) used AND whether they are one- or two-sided<br>*Only common tests should be described solely by name; describe more complex techniques in the Methods section.*
☒	☐	A description of all covariates tested
☒	☐	A description of any assumptions or corrections, such as tests of normality and adjustment for multiple comparisons
☐	☒	A full description of the statistical parameters including central tendency (e.g. means) or other basic estimates (e.g. regression coefficient) AND variation (e.g. standard deviation) or associated estimates of uncertainty (e.g. confidence intervals)
☐	☒	For null hypothesis testing, the test statistic (e.g. *F*, *t*, *r*) with confidence intervals, effect sizes, degrees of freedom and *P* value noted<br>*Give P values as exact values whenever suitable.*
☒	☐	For Bayesian analysis, information on the choice of priors and Markov chain Monte Carlo settings
☒	☐	For hierarchical and complex designs, identification of the appropriate level for tests and full reporting of outcomes
☒	☐	Estimates of effect sizes (e.g. Cohen's *d*, Pearson's *r*), indicating how they were calculated

Our web collection on statistics for biologists contains articles on many of the points above.

Software and code

Policy information about availability of computer code

Data collection	Intensity of immunoreactive band signal from immunoblots was measured using Image Lab 6.1 software (Bio-Rad Laboratories). Cell viability assays were measured on an Perkin Elmer Envision Plate reader. RT-PCR experiments were run on an QuantStudio 6 Pro-RT-PCR System.<br>For chemical compounds characterization we used: a) 1H and 13C NMR spectra were recorded on an Agilent DD2 500 (500 MHz 1H; 125 MHz 13C) or Agilent DD2 600 (600 MHz 1H; 150 MHz 13C) or Agilent DD2 400 (400 MHz 1H; 100 MHz 13C) spectrometers, and run the analysis at room temperature. b) Mass spectra data were obtained using a LC-MS Waters Xevo G2-XS QTof Quadrupole Time-of-Flight Mass Spectrometer.
Data analysis	Graphpad PRISM Version 8.4.2<br>Crystal structure molecular visualization was performed using the Molecular Operating Environment software (MOE-2019.0102).

For manuscripts utilizing custom algorithms or software that are central to the research but not yet described in published literature, software must be made available to editors and reviewers. We strongly encourage code deposition in a community repository (e.g. GitHub). See the Nature Research guidelines for submitting code & software for further information.

Data

Policy information about availability of data

All manuscripts must include a data availability statement. This statement should provide the following information, where applicable:

- Accession codes, unique identifiers, or web links for publicly available datasets
- A list of figures that have associated raw data
- A description of any restrictions on data availability

Linker positioning was determined using structure of vemurafenib bound to BRAF (PDB:3OG7). Source data are provided with this paper

Field-specific reporting

Please select the one below that is the best fit for your research. If you are not sure, read the appropriate sections before making your selection.

☒ Life sciences ☐ Behavioural & social sciences ☐ Ecological, evolutionary & environmental sciences

For a reference copy of the document with all sections, see nature.com/documents/nr-reporting-summary-flat.pdf

Life sciences study design

All studies must disclose on these points even when the disclosure is negative.

Sample size	No statistical methods were used to predetermine sample size. Sample sizes were selected based on our previous studies.
Data exclusions	Some western blot lanes showing duplicated samples were excluded. PDX Ru37 mice experiments terminated due to COVID working restrictions were excluded.
Replication	All in vitro or cell based experiments were performed two or more independent times. All attempts at replication of experimental results were successful. For mice studies: Mice bearing xenografts of cancer cells were treated with either vehicle or drug to determine the effect on tumor growth. Tumor sizes are represented by mean ± SD in the graphs (n = 3). The replicates for tumor growth measurements indicate biological replicates.
Randomization	For in vivo tumor growth experiments: implanted mice were treated in a random manner with vehicle, or drug treatment. Randomization was not relevant to the remaining cell based or biochemical experiments.
Blinding	Investigators were not blinded to the nature of their samples during data collection and analysis. Experiments were designed based on preliminary results.

Reporting for specific materials, systems and methods

We require information from authors about some types of materials, experimental systems and methods used in many studies. Here, indicate whether each material, system or method listed is relevant to your study. If you are not sure if a list item applies to your research, read the appropriate section before selecting a response.

Materials & experimental systems

n/a	Involved in the study	
☐	☒	Antibodies
☐	☒	Eukaryotic cell lines
☒	☐	Palaeontology and archaeology
☐	☒	Animals and other organisms
☒	☐	Human research participants
☒	☐	Clinical data
☒	☐	Dual use research of concern

Methods

n/a	Involved in the study	
☒	☐	ChIP-seq
☒	☐	Flow cytometry
☒	☐	MRI-based neuroimaging

Antibodies

Antibodies used

The following antibodies were used in this study: Primary antibodies from Cell-Signaling
- anti-V5 (no. 13202), Dilution 1:1000
- anti-BRAF (no. 14814s), Dilution 1:1000
- anti-CRAF (no.53745), Dilution 1:1000
- anti-p202/p204-ERK1/2 (p-ERK1/2) (no. 4370), Dilution 1:3000
- anti-ERK1/2 (no.4696s) , Dilution 1:1000
- anti-MEK1/2 (no. 8727), Dilution 1:1000
- anti-p217/p221-MEK1/2 (p-MEK1/2) (no. 9154) ,Dilution 1:1000
- anti-GAPDH (no.2118S), Dilution 1:1000
- anti-VHL (no. 68547) Dilution 1:1000
- anti-phospho-CRaf (Ser338) (no. 9427) Dilution 1:1000
- anti-HA (no. 2367), Dilution 1:1000
- anti-ARAF(no. 75804s),Dilution 1:1000
- anti-ubiquitin (no. 43124) , Dilution 1:500
- anti-RBX-1 (no. 11922S), Dilution 1:1000
- anti-p-HER2/ErbB2 (Y1221/1222) (6B12) (no. 2243S) Dilution 1:1000

Primary antibody from Millipore: anti-tubulin (16-232); Dilution 1:3000
Primary antibody from Invitrogen: anti-Cullin 2(700179), Dilution 1:1000

Primary antibody from Lifespan Biosciences: anti-KRAS (LS-C175665), Dilution 1:1000
Secondary antibodies were from ThermoFisher:
• anti-rabbit HRP (31460) Dilution 1:50000
• anti-mouse HRP (31444) Dilution 1:10000

Validation

All the antibodies used in this study were purchased from commercial sources and had been validated by their respective manufacturer for immunoblotting and/or immunoprecipitation (the applications used in this study) of the human isoform of their cognate antigens.

Cell Signaling Validation Statement:
To ensure our antibodies will work in your experiment, we adhere to the Hallmarks of Antibody Validation™, six complementary strategies that can be used to determine the functionality, specificity, and sensitivity of an antibody in any given assay. CST adapted the work by Uhlen, et. al., ("A Proposal for Validation of Antibodies." Nature Methods (2016)) to build the Hallmarks of Antibody Validation, based on our decades of experience as an antibody manufacturer and our dedication to reproducible science.
• Binary Model: Antibody signal is measured in model systems with known presence/absence of target signal. Includes wild-type vs. genetic knockout, targeted induction or silencing.
• Ranged Expression: Antibody signal strength is measured in cell lines or tissues representing a known continuum of target expression levels. Includes siRNA and heterozygous knockout assays.
• Orthogonal Data: Antibody signal is correlated to target expression in model systems measured using antibody independent assays. Includes mass spectrometry and in situ hybridization.
• Multiple Antibodies: Antibody signal is compared to the signal observed using antibodies targeting nonoverlapping epitopes of the target. Includes IP, ChIP, and ChIP-seq.
• Heterologous Expression: Antibody signal is evaluated in cell lines following heterologous expression of native (or mutated) target protein.
• Complementary Assays: Antibody specificity may be validated using complementary assays. Includes competitive ELISA, peptide dot blots, peptide blocking, or protein array

The following antibodies were used in this study: Primary antibodies from Cell-Signaling
• anti-V5 (no. 13202), Dilution 1:1000
https://www.cellsignal.com/datasheet.jsp?productId=13202&images=1
• anti-BRAF (no. 14814s), Dilution 1:1000
https://www.cellsignal.com/datasheet.jsp?productId=14814&images=1
• anti-CRAF (no.53745), Dilution 1:1000
https://www.cellsignal.com/datasheet.jsp?productId=53745&images=1
• anti-p202/p204-ERK1/2 (p-ERK1/2) (no. 4370), Dilution 1:3000
https://www.cellsignal.com/datasheet.jsp?productId=4370&images=1
• anti-ERK1/2 (no.4696s) , Dilution 1:1000
https://www.cellsignal.com/datasheet.jsp?productId=4696&images=1
• anti-MEK1/2 (no. 8727) Dilution 1:1000
https://www.cellsignal.com/datasheet.jsp?productId=8727&images=1
• anti-p217/p221-MEK1/2 (p-MEK1/2) (no. 9154) Dilution 1:1000
https://www.cellsignal.com/datasheet.jsp?productId=9154&images=1
• anti-GAPDH (no.2118S) Dilution 1:1000
https://www.cellsignal.com/datasheet.jsp?productId=2118&images=1
• anti-VHL (no. 68547) Dilution 1:1000
https://www.cellsignal.com/datasheet.jsp?productId=68547&images=1
• anti-phospho-CRaf (Ser338) (no. 9427) Dilution 1:1000
https://www.cellsignal.com/datasheet.jsp?productId=9427&images=1
• anti-HA (no. 2367), Dilution 1:1000
https://www.cellsignal.com/datasheet.jsp?productId=2367&images=1
• anti-ARAF(no. 75804s)
https://www.cellsignal.com/datasheet.jsp?productId=75804&images=1
• anti-ubiquitin (no. 43124) , Dilution 1:500
https://www.cellsignal.com/datasheet.jsp?productId=43124&images=1
• anti-RBX-1 (no. 11922S), Dilution 1:1000
https://www.cellsignal.com/datasheet.jsp?productId=11922&images=1
• anti-p-HER2/ErbB2 (Y1221/1222) (6B12) (no. 2243S) Dilution 1:1000
2243S

Primary antibody from Millipore: anti-tubulin (16-232); Dilution 1:3000
Routinely evaluated by Flow Cytometry.Flow Cytometry:
0.2 µg of this lot detected α-Tubulin in fixed and permeabilized rat L6, Jurkat, and A431 cells
Primary antibody from Invitrogen: anti-Cullin 2(700179), Dilution 1:1000
Knockdown
This Antibody was verified by Knockdown to ensure that the antibody binds to the antigen stated.

Primary antibody from Lifespan Biosciences: anti-KRAS (LS-C175665), Dilution 1:1000
Validated in our previous publication and PubMed: 28706291 PMC: PMC5519984
Secondary antibodies were from ThermoFisher:

• anti-rabbit HRP (31460) Dilution 1:50000
https://www.thermofisher.com/order/genome-database/dataSheetPdf?producttype=antibody&productsubtype=antibody_secondary&productId=31460&version=125
• anti-mouse HRP (31444) Dilution 1:10000
https://www.thermofisher.com/order/genome-database/dataSheetPdf?producttype=antibody&productsubtype=antibody_secondary&productId=31444&version=125

Eukaryotic cell lines

Policy information about cell lines

Cell line source(s)	SK-MEL-28 (E-MEM), A-431 (D-MEM), NIH-3T3 (D-MEM) and SKBR3(RPMI) cells was obtained from ATCC. Inducible 293-Trex (HEK-293 TRex) (DMEM), CAL-12T (DMEM), H1666 (RPMI), A375 (DMEM)and SK-MEL-30 cells (RPMI) were obtained from Arvinas. Remainder of the cells lines were obtained from other labs: We thank the Kupfer Laboratory for HCT-116 cells (DMEM), Slack Laboratory for H23 cells, A. Houghton and P. Chapman Labs for SKMEL 246 cells (DMEM) and SK-MEL-239 and SK-MEL-239 C4 cells (DMEM;1 µM vemurafenib), Joyce Lui Laboratory for OVCAR-8 cells, and the Trevor Bivona Laboratory for HCC364 vr1 cells (10 µM vemurafenib). Inducible expression NIH3T3 cells were maintained in DMEM;50 µg ml−1 hygromycin and 0.2 µg ml−1 puromycin). All media was supplemented with 10% fetal bovine serum and 1% penicillin–streptomycin and grown in a humidified incubator at 37 °C and 5% CO_2.
Authentication	The cell lines purchased from ATCC were purchased as 'Certified Reference Material' stocks. Cell lines received from other labs were not verified. ATCC authenticates cell lines using morphology, karyotyping and STR profiling.
Mycoplasma contamination	The cell lines used in this study tested negative for mycoplasma contamination according to the MycoAlert test kit from Lonza.
Commonly misidentified lines (See ICLAC register)	None of the cell lines used in this study are listed in the current version (v.9) of the ICLAC register.

Animals and other organisms

Policy information about studies involving animals; ARRIVE guidelines recommended for reporting animal research

Laboratory animals

All our mouse studies are conducted in accordance with protocols approved by the Institutional Animal Care and Use Committee (IACUC) of Memorial Sloan Kettering Cancer Center (MSKCC).
Memorial Sloan-Kettering Cancer Center's animal care and use program is administered by the Research Animal Resource Center (RARC). The program has been fully accredited by the Association of Assessment and Accreditation of Animal Care, International (AAALAC) since 1967, is registered with the USDA, and has an approved assurance on file with the Office of Laboratory Animal Welfare, NIH (OLAW). RARC is staffed by board-certified laboratory animal veterinarians and pathologists, veterinary and animal care technicians, management, and administrative support staff. Veterinary staff is available 24 hours a day, 7 days a week to address emergencies. The program is supported by the Laboratory of Comparative Pathology which provides anatomic and clinical pathologic evaluation of animals, tissues, and fluids in support of animal health and the use of animal models.

The animal resource program is housed in three state-of-the-art facilities occupying a total of 62,500 net ft2 of usable space. All vivaria contain barrier rodent housing facilities. Specialized facilities for the use of animal models exposed to biological and hazardous chemical agents and for conducting surgical procedures in large and small animals are available.

Female athymic nu/nu mice, 6-8 weeks old .
temperature: 72 degrees F
humidity: 50% relative humidity
lighting: 12 hours on starting at 7 am/12 hours off starting at 7 pm

the mice have access to food and water ad libitum

Wild animals	Study did not include wild animals
Field-collected samples	Study did not include field-collected samples

Ethics oversight

Memorial Sloan Kettering Cancer Center Antitumor Assessment Core Facility oversaw and approved the animal study. Members of the Core Facility are listed below:
Elisa de Stanchina
Juan (Jane) Qiu
Huiyong Zhao
Nica Aquino
Amber Bahr
Qing Chang
Xiaoping Chen
Ana Crawford
Deborah Fidele

Note that full information on the approval of the study protocol must also be provided in the manuscript.
