# Supplementary material for: Mutant-selective degradation by BRAF-targeting PROTACs
Source: Nat Commun. 2021 Feb 10;12:920. doi: 10.1038/s41467-021-21159-7 (PMC7876048; doi:10.1038/s41467-021-21159-7)
Supplement: Supplementary file 2 — Supplementary Information [file 41467_2021_21159_MOESM2_ESM.pdf]

Supplementary

Mutant-selective Degradation by BRAF-targeting PROTACs

Shanique Alabi[1], Saul Jaime-Figueroa[2], Zhan Yao[3], Yijun Gao[3], John Hines[2], Kusal T.G. Samarasinghe[2], Lea Vogt[2], Neal Rosen[3], Craig M. Crews[1,2,4*]

[1]Department of Pharmacology, 333 Cedar Street, New Haven, CT, USA

[2]Molecular, Cellular, and Developmental Biology, Yale University, 260 Whitney Avenue, New Haven, CT, 06511,USA

[3]Program in Molecular Pharmacology, Memorial Sloan Kettering Cancer Center, New York, New York 10065, USA

[4]Department of Chemistry, Yale University, 225 Prospect Street, New Haven CT, USA.

*corresponding author:craig.crews@yale.edu

Supplementary Figures

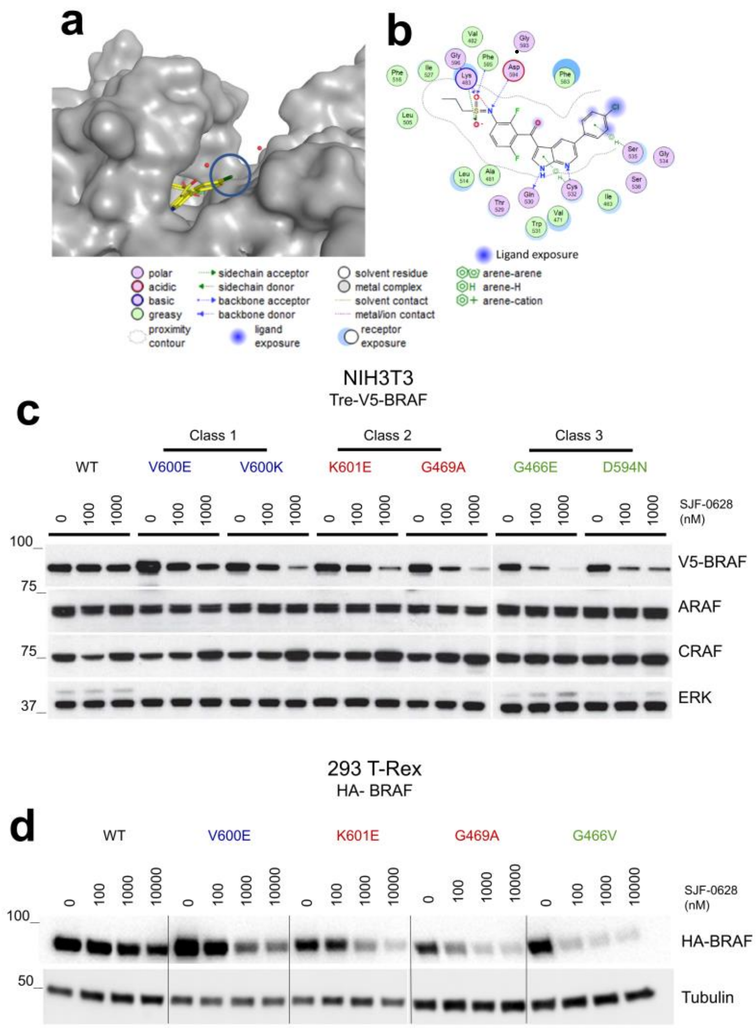

Supplementary Figure 1. Vemurafenib based PROTAC, SJF-0628, induces mutant selective degradation of BRAF a, Crystal structure of BRAF^{V600E} in complex with vemurafenib (PDB: 3OG7[1]) **b**, Ligand interactions diagram showing important BRAF: vemurafenib interactions and solvent exposure. **c**, Inducible NIH3T3 cells expressing indicated V5-BRAF (doxycycline 500 ng/mL, 24 hours) treated with increasing amounts of SJF-0628 for 24 hours. **d**, 293 T-Rex cells (doxycycline 20 ng/mL, 24 hours) expressing indicated BRAF isoforms treated with increasing concentrations of SJF-0628 shows mutant selective degradation. Source data are provided as a Source Data file.

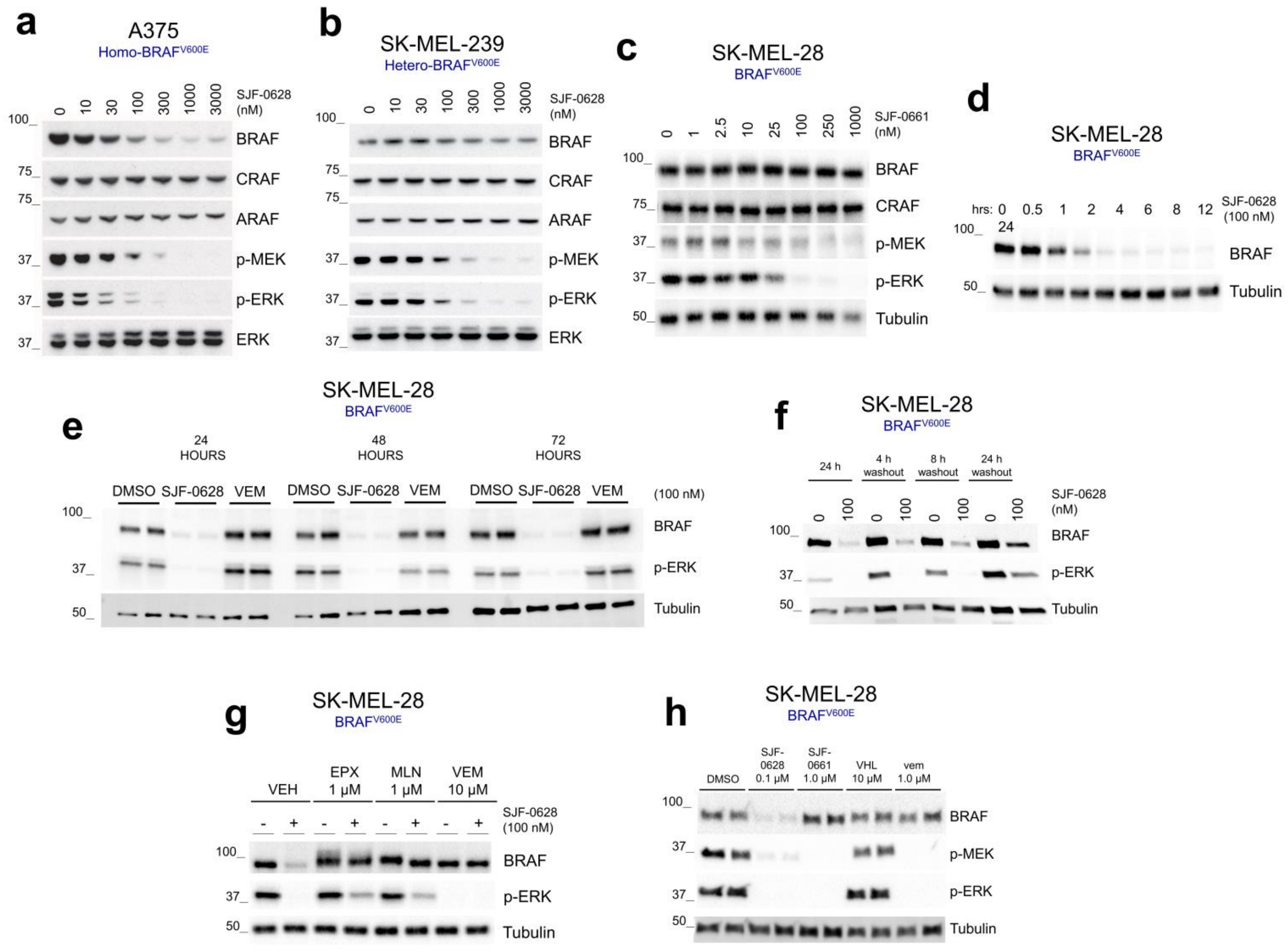

Supplementary Figure 2. SJF-0628 induces a sustained and efficient degradation of BRAF^{V600E} via the proteasome a, Treatment of A375 (homozygous BRAF^{V600E}) cells with SJF-0628 shows BRAF^{V600E} degradation and inhibition of MEK and ERK phosphorylation. **b**, Treatment of SK-MEL-239 (heterozygous BRAF^{V600E}) cells with SJF-0628 shows minimal BRAF degradation but marked inhibition of MEK and ERK phosphorylation. **c**,SK-MEL-28 cells treated with negative control epimer, SJF-0661, does not affect BRAF levels but inhibits ERK signaling. **d**, Representative immunoblot of SJF-0628 time course (100 nM) at indicated times in SK-MEL-28 cells (plotted in Fig. 1e) shows maximal degradation within 4 hours. **e**, Treatment of SK-MEL-28 cells with 100 nM of SJF-0628, and vemurafenib for indicated times shows sustained degradation and inhibition of MAPK signaling (n=2 biologically independent samples). **f**, SK-MEL-28 cells were treated with 100 nM for 24 hours, washed 3 times with DPBS and replenished with fresh media. Cells were then lysed either 4, 8 or 24 hours after media removal to determine level of BRAF and p-ERK protein level recovery. **g**, SK-MEL-28 cells treated with a proteasome inhibitor (EPX = epoxomicin), a neddylation inhibitor (MLN = MLN4924), or excess vemurafenib (VEM) for 2 hours, then subsequently treated with DMSO or PROTAC for 8 hours. **h**, SK-

MEL-28 cells treated with indicated compound for 6 hours. VHL ligand alone does not cause MAPK inhibition (n=2 biologically independent samples). Source data are provided as a Source Data file.

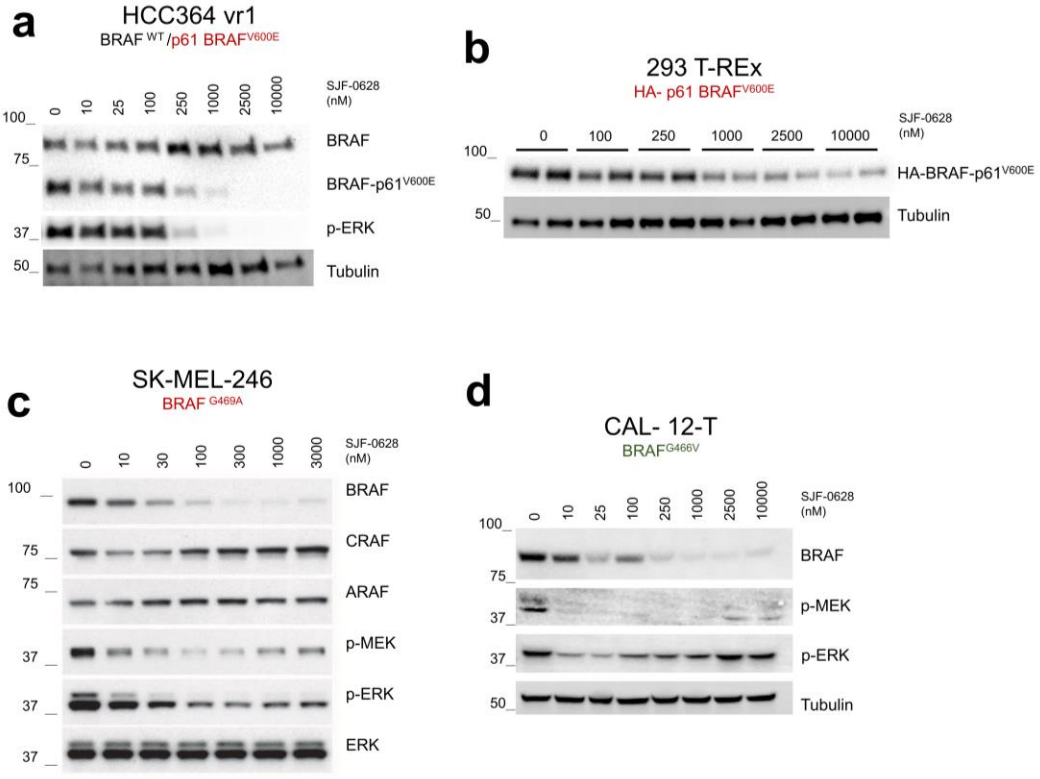

Supplementary Figure 3. SJF-0628 induces degradation of acquired and intrinsic vemurafenib-resistant BRAF mutants a, HCC364 vr1 ($BRAF^{WT}$, p61-$BRAF^{V600E}$) selectively induces degradation of p61-$BRAF^{V600E}$ and spares $BRAF^{WT}$. **b**, SJF-0628 treatment in 293 T-Rex cells expressing HA-p61-$BRAF^{V600E}$ shows dose dependent decrease in HA-p61-$BRAF^{V600E}$ protein levels (n=2 biologically independent samples). **c**, SK-MEL-246 (Class 2, BRAF ^{G469A}) cells treated with increasing amount of SJF-0628 shows degradation of BRAF and inhibition of ERK signaling. **d**, CAL-12-T cells (homozygous $BRAF^{G466V}$) treated with SJF-0628 shows BRAF degradation, but incomplete suppression of ERK signaling. Source data are provided as a Source Data file.

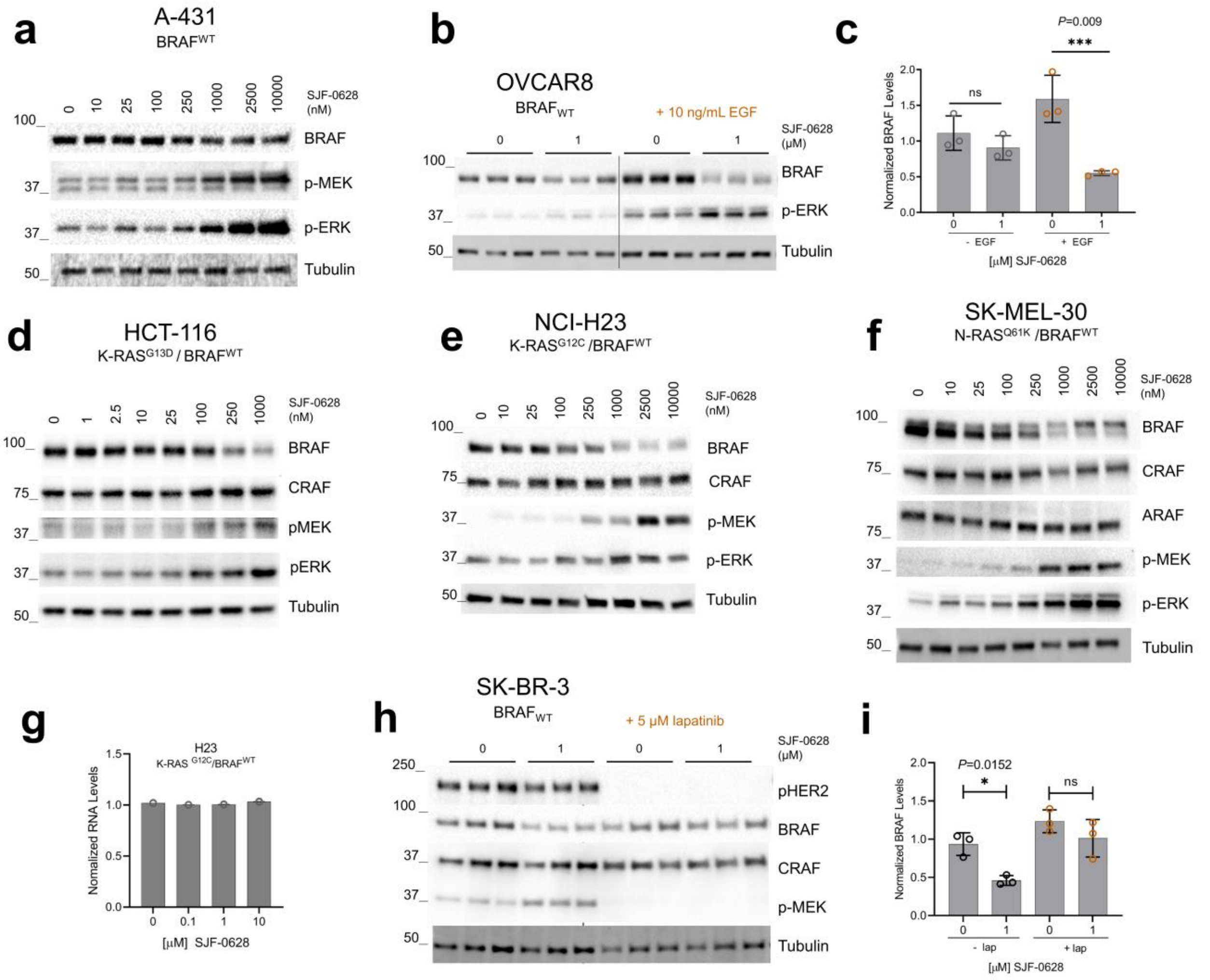

Supplementary Figure 4. BRAF^{WT} is sensitized to PROTAC induced degradation in the presence of activated upstream drivers

a, Treatment of A-431 cells (HER1 amplification) which expresses BRAF^{WT} and RAS^{WT} treated with increasing amounts of SJF-0628 show some degradation of BRAF^{WT}(~30%). **b**, Serum-starved OVCAR8 cells stimulated with 10 ng/mL of EGF promotes SJF-0628 induced degradation of BRAF^{WT} (mean ± SD, n=3 biologically independent samples). **c,** quantification of BRAF levels in (**b**) *P* value calculated by one sided ANOVA. **d**-**f**, SJF-0628 treatment in HCT116, H23, and SK-MEL-30 cells bearing a RAS mutation shows 40%-60% degradation of BRAF^{WT} at high concentrations and paradoxical activation of MAPK signaling. **g,** Quantitative real time PCR of H23 cells treated with SJF-0628 for 20 hours (mean ± SD, n=3 biologically independent samples). **h**, SKBR3 cells pretreated with lapatinib hinders PROTAC induced degradation of BRAF^{WT} **i,** quantification of BRAF levels in **(h)** (mean ± SD, n=3 biologically independent samples) *P* value calculated by one sided ANOVA. Source data are provided as a Source Data file.

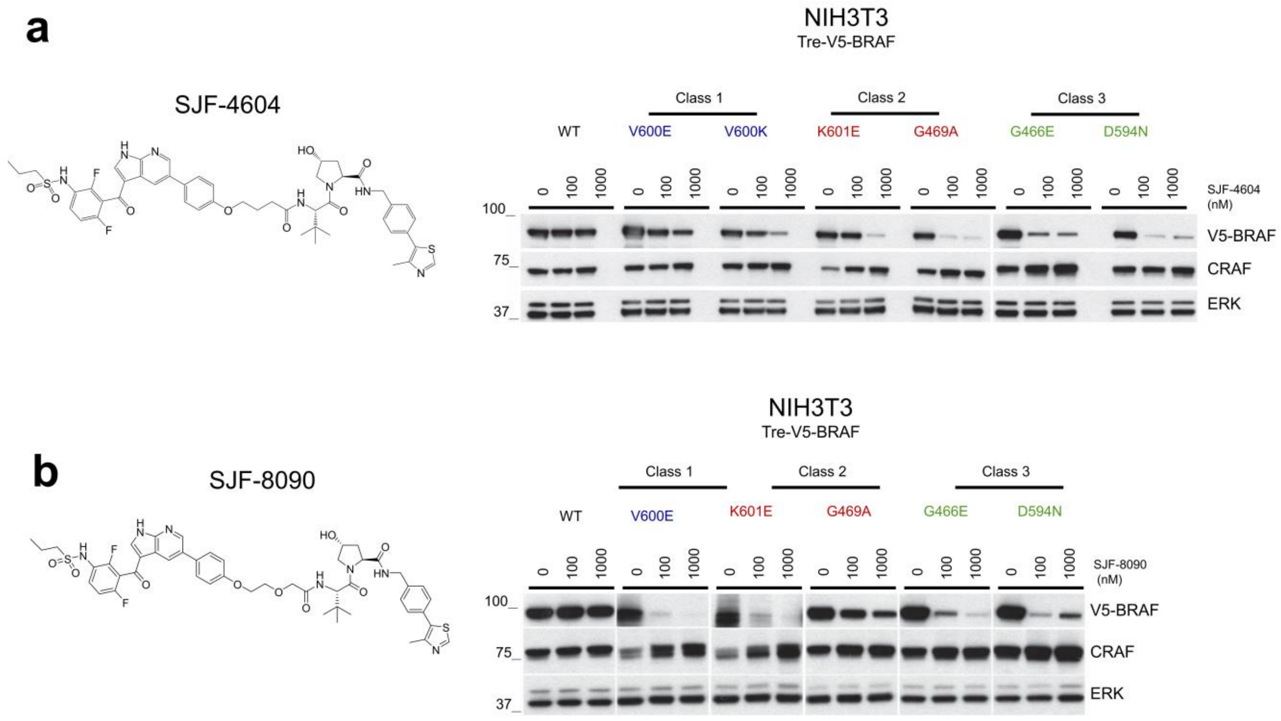

Supplementary Figure 5. Vemurafenib based PROTACs spare BRAF^{WT} despite varied linker length and composition a-b, Structures and results of vemurafenib based PROTACs SJF-4604 and SJF-8090 treatment in inducible NIH3T3 cells expressing indicated BRAF protein for 24 hours. Both PROTACs show mutant selective degradation. Source data are provided as a Source Data file.

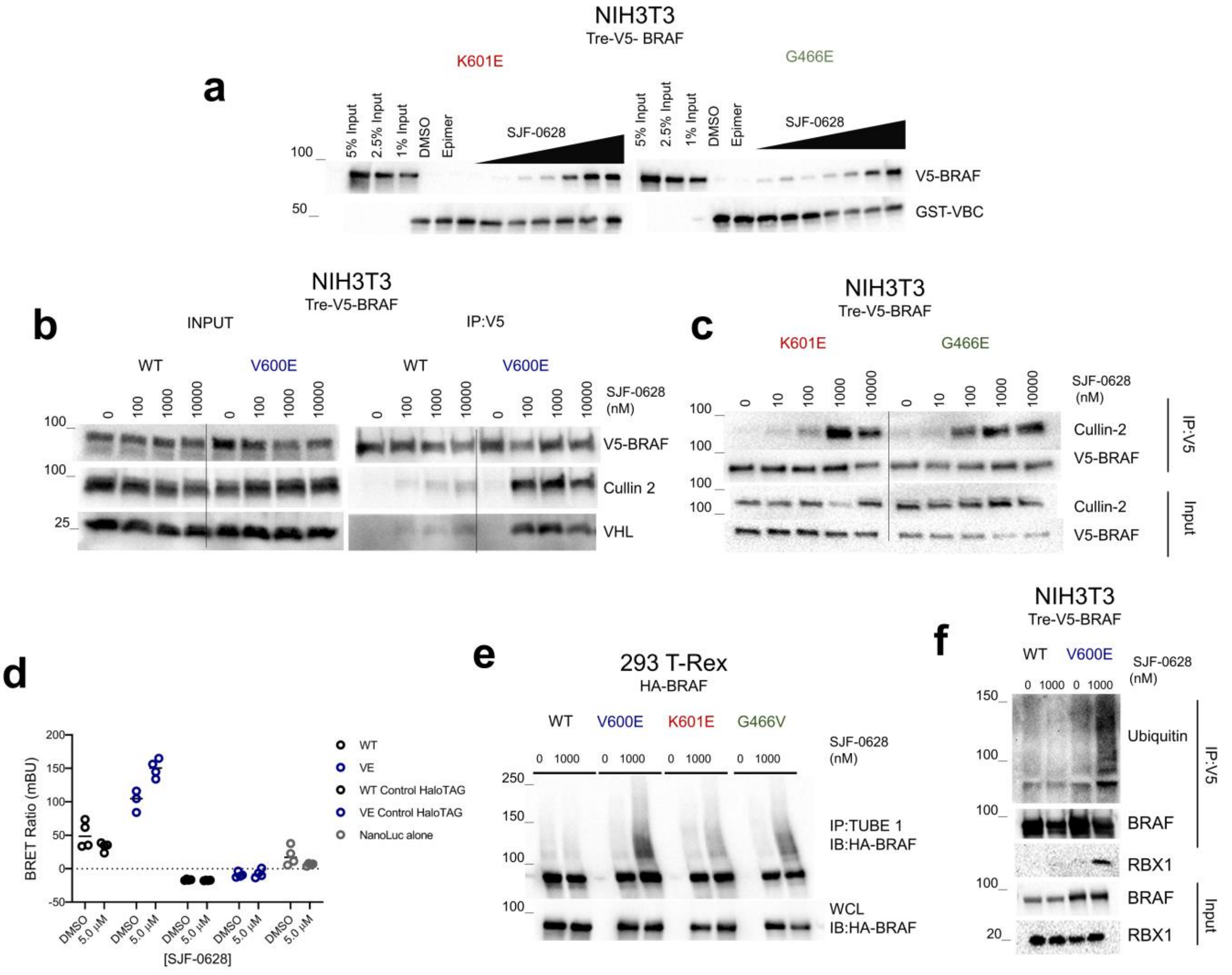

Supplementary Figure 6. All three mutant BRAF classes bind SJF-0628 and form a stable ternary complex with VHL which promote its ubiquitination a, Cell lysate-based trimer assay of VBC immobilized on glutathione beads incubated with NIH3T3 cell lysates expressing BRAF^{K601E} and BRAF^{G466E} with vehicle, 500 nM SJF-0661, or increasing amounts of SJF-0628. **b**, V5-BRAF immunoprecipitation in NIH3T3 cells (WT and V600E) stably expressing human VHL treated with increasing concentrations SJF-0628 for 45 mins. **c,** V5-BRAF immunoprecipitation in NIH3T3 cells expressing BRAF^{K601E} or BRAF^{G466E} treated with SJF-0628 for 1 hour. **d**, Control NanoBRET experiments. WT and VE= transfection of BRAF(WT or V600E)-NanoLuc and VHL-HaloTag. WT Control HaloTag =Transfection of nanoLUC-BRAF^{WT} with HaloTag alone (without VHL); VE Control HaloTag = Transfection of nanoLUC-BRAF^{V600E} with HaloTag alone (without VHL); NanoBRET alone= Transfection of NanoLuc alone (without BRAF) with VHL-HaloTag (mean ± SD, n=4 biologically independent experiments). **e**, Tandem Ubiquitin Binding Entities 1 (TUBE1) Assay. Pulldown of tetra-ubiquitinated protein in 293 T-REx cells expressing indicated BRAF treated with SJF-0628 for 1 hour. Immunoblot for HA-BRAF. **f**, V5-BRAF immunoprecipitation in NIH3T3 cells expressing BRAF^{WT} and

$BRAF^{V600E}$ treated with SJF-0628 for 1 hour. Pulldown shows SJF-0628 induced ubiquitination of $BRAF^{V600E}$ but not $BRAF^{WT}$. Source data are provided as a Source Data file.

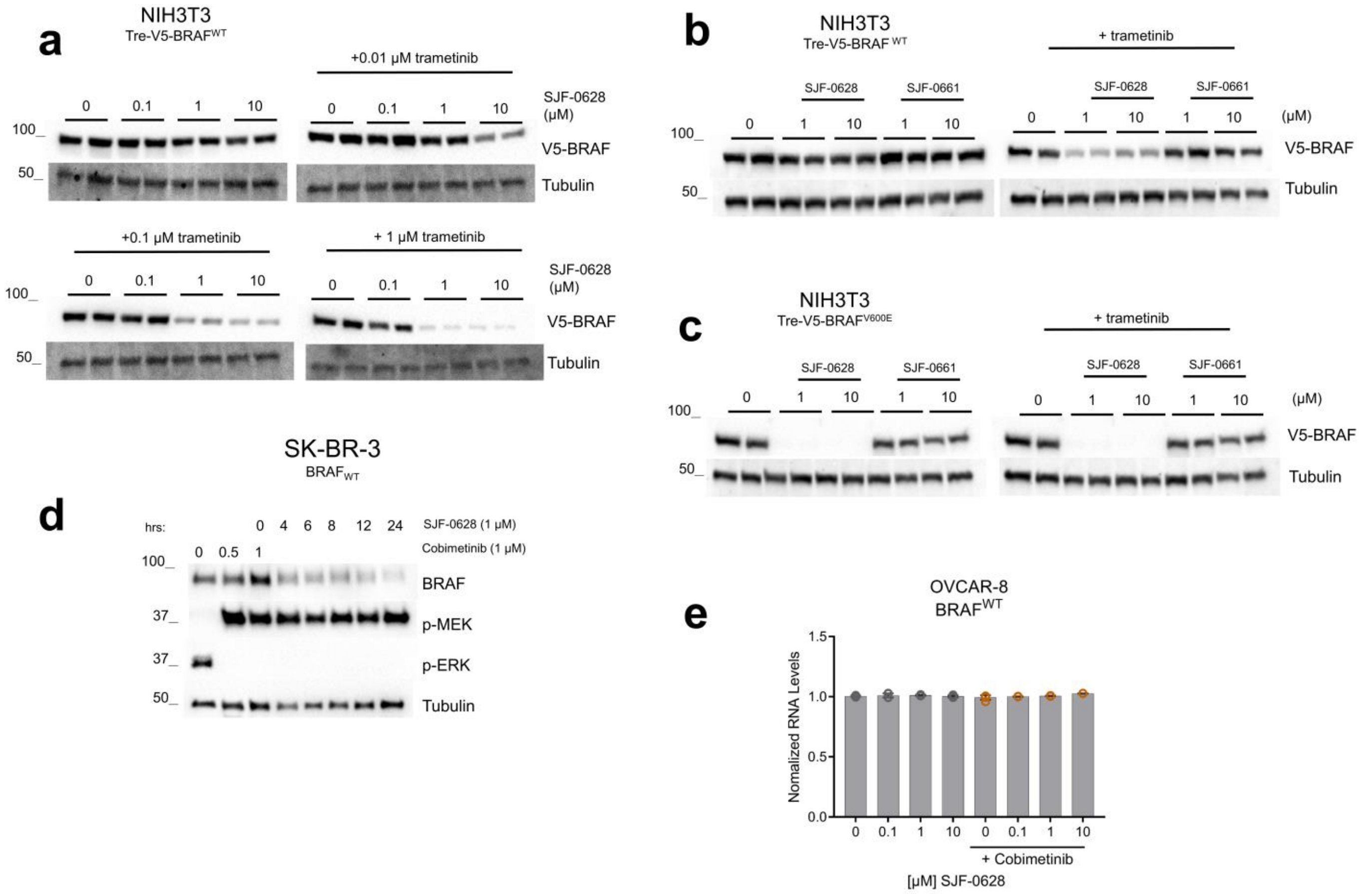

Supplementary Figure 7. Trametinib or cobimetinib pre-treatment promotes dose and time dependent degradation of BRAF^{WT} a, NIH3T3 cells expressing inducible V5-BRAF^{WT} pre-treated with increasing concentrations of trametinib and subsequently treated with increasing concentrations of SJF-0628 (n=2 biologically independent samples). **b-c**, NIH3T3 cells expressing BRAF^{WT} and BRAF^{V600E} pre-treated with trametinib followed by SJF-0628 or SJF-0661 treatment (n=2 biologically independent samples). **d,** Time course of SK-BR-3 cells pre-treated with 1 μM of cobimetinib for 1 hour then treated with 1 μM SJF-0628. **e**, mRNA expression changes of BRAF pre-treated with 1 μM of cobimetinib for 1 hour, then treated with 1 μM SJF-0628 for 20 hours (mean ± SD, n=3 biologically independent samples). Source data are provided as a Source Data file.

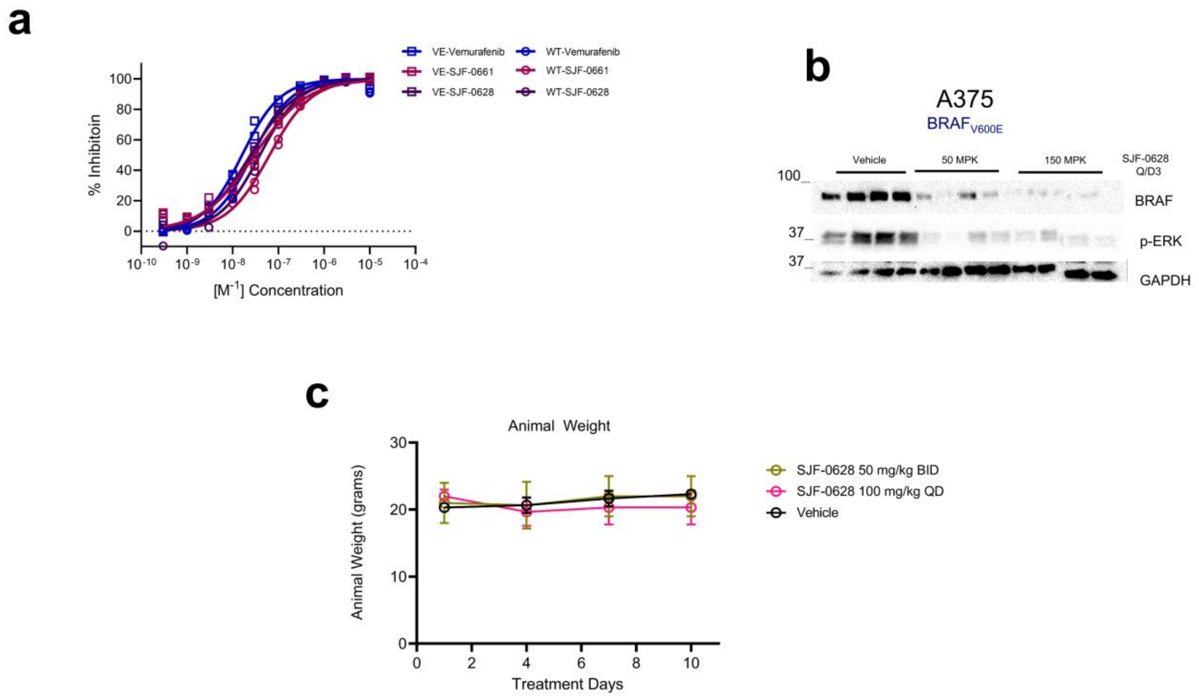

Supplementary Figure 8. MAPK inhibitors that increase BRAF kinase activity promote SJF-0628 induced $BRAF^{WT}$ degradation a, Immunoprecipitation of V5-$BRAF^{WT}$ from NIH3T3 cells treated with 1µM of the indicated MAPK pathway inhibitor. Cobimetinib treatment showed minimal BRAF association with MEK, but increased RAF dimerization while GDC-0623 showed minimal RAF dimerization, and increased BRAF:MEK association. **b-c**, OVCAR-8 cells and SK-BR-3 cells pre-treated with GDC-0623 and cobimetinib (500nM, 3 hours) then treated with SJF-0628 for 20 hours. Source data are provided as a Source Data file.

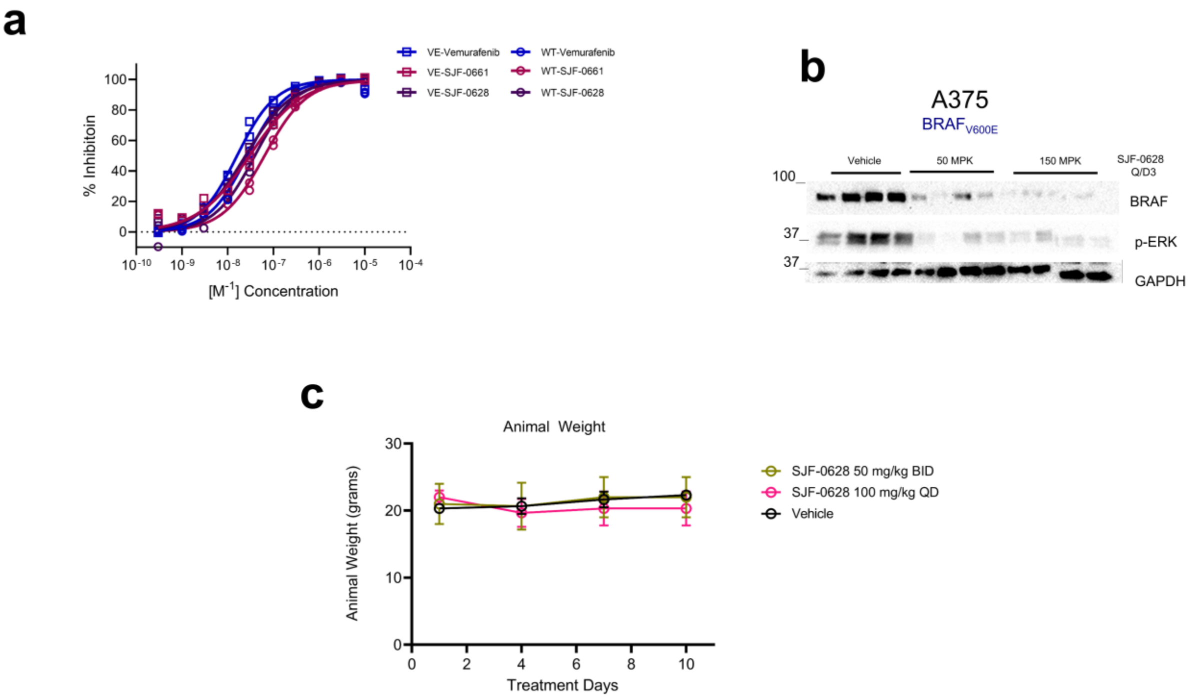

Supplementary Figure 9. SJF-0628 inhibits $BRAF^{WT}$ and $BRAF^{V600E}$ with a similar affinity and induces degradation of $BRAF^{V600E}$ *in vivo* a, $BRAF^{WT}$ and $BRAF^{V600E}$ binding affinity and curves for ELISA kinase inhibition assay with SJF-0628, SJF-0661 and vemurafenib IC_{50} values in Supplementary Table 2 (n=2 biologically independent experiments). **b,** $BRAF^{V600E}$ degradation in A375 xenograft in female Balb/c nude mice treated with indicated concentrations of SJF-0628, QDx3. Tumors were harvested 8 hours after last treatment (n=4 biologically independent samples). **c,** Average mice body weight in SK-MEL-246 efficacy study treated with SJF-0628 (mean ± SD, n=3 biologically independent animals). Source data are provided as a Source Data file.

Supplementary Tables

Beta_Tub_F:	5'-TGGACTCTGTTCGCTCAGGT-3
Beta_Tub_R:	5'-TGCCTCCTTCCGTACCACAT-3'
BRAF_F:	5'-GAGGCGTCCTTAGCAGAGAC-3'
BRAF_R:	5'-AAGGAGACGGACTGGTGAGAAF-3'

Supplementary Tables 1 RT-PCR Primers Used

Kinase	IC_{50} (M)		
	**vemurafenib**	**SJF-0628**	**SJF-0661**
BRAF ^{V600E}	1.60E-08	2.44E-08	3.00E-08
BRAF ^{WT}	2.70E-08	3.92E-08	6.40E-08

Supplementary Table 2 IC_{50} values from Elisa Kinase inhibition Assay

Supplementary Methods

Supplementary Methods

Chemical syntheses of PROTACs

1. General experimental

General comments. Unless otherwise indicated, common reagents or materials were obtained from commercial source and used without further purification. Tetrahydrofuran (THF), dimethylformamide (DMF), and Dichloromethane (DCM) were dried by a PureSolv™ solvent drying system. Flash column chromatography was performed using silica gel 60 (230-400 mesh). Analytical (TLC) and preparative (PTLC) thin layer chromatography was carried out on Merck silica gel plates with QF-254 indicator and visualized by UV or iodine. ^{1}H and ^{13}C NMR spectra were recorded on an Agilent DD$_2$ 500 (500 MHz ^{1}H; 125 MHz ^{13}C) or Agilent DD$_2$ 600 (600 MHz ^{1}H; 150 MHz ^{13}C) or Agilent DD$_2$ 400 (400 MHz ^{1}H; 100 MHz ^{13}C) spectrometer at room temperature. Chemical shifts were reported in ppm relative to the residual $CDCl_3$ (δ 7.26 ppm ^{1}H; δ 77.00 ppm ^{13}C), CD_3OD (δ 3.31 ppm ^{1}H; δ 49.00 ppm ^{13}C), or d^6-DMSO (δ 2.50 ppm ^{1}H; δ 39.52 ppm ^{13}C). NMR chemical shifts were expressed in ppm relative to internal solvent peaks, and coupling constants were measured in Hz. (bs = broad signal). Mass spectra were obtained using electrospray ionization (ESI) on a time of flight (TOF) mass spectrometer. . Compounds **1**[2], **VHL** ligands[3] 5 and 6 were prepared according with the literature or acquired commercially.

2. Synthesis of PROTACs SJF-0628 and SJF-0661

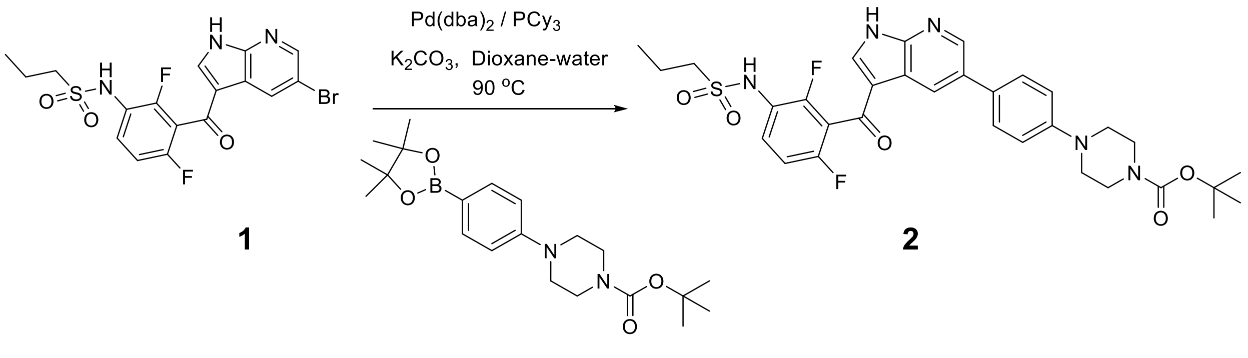

***tert*-Butyl 4-(4-(3-(2,6-difluoro-3-(propylsulfonamido)benzoyl)-1H-pyrrolo[2,3-b]pyridin-5-yl)phenyl) -piperazine-1-carboxylate (2)**. To a solution of N-[3-(5-bromo-1H-pyrrolo[2,3-b]pyridine-3-carbonyl)-2,4-difluoro-phenyl]propane-1-sulfonamide (1) (70.8 mg, 0.155 mmol) in Dioxane (6 ml) was added tert-butyl 4-(4-(4,4,5,5-tetramethyl-1,3,2-dioxaborolan-2-yl)phenyl)piperazine-1-carboxylate (60.0 mg, 0.155 mmol), K_2CO_3 (64.2 mg, 0.465 mmol), Tricyclohexyl phosphine (4.33 mg, 0.0155

mmol)and water (2 mL). Then the reaction mixture was de-gassed under vacuum and purged with argon (5x), $Pd(dba)_2$ (4.44 mg, 0.00773 mmol) was added into and the reaction mixture was heated at 90 °C for 3 h. By TLC small amounts of SM (Hex:EtOAc, 3:7), the reaction mixture was filtered in vacuum over a celite pad, filtrate was poured into an aqueous saturated solution of NaCl (20 mL) and the product was extracted with EtOAc (2x20 mL). The EtOAc layers were combined, dried (Na_2SO_4) and concentrated in vacuum. The crude material was diluted in DCM and purified by flash chromatography (SiO_2-12g, Hexane:EtOAc, 9:1 to 100% EtOAc in 15 min) to give 82 mg (83%) of product. ^{1}H NMR (500 MHz, DMSO-d6) δ 12.92 (bs, 1H), 9.76 (bs, 1H), 8.66 (d, J = 2.2 Hz, 1H), 8.55 (s, 1H), 8.19 (s, 1H), 7.75 – 7.46 (m, 3H), 7.28 (t, J = 8.7 Hz, 1H), 7.10 (d, J = 8.7 Hz, 2H), 3.49 (t, 4H), 3.19 (t, J = 5.2 Hz, 4H), 3.16 – 3.05 (m, 2H), 1.74 (h, J = 7.5 Hz, 2H), 1.43 (s, 9H), 0.96 (t, J = 7.4 Hz, 3H). ^{13}C NMR (151 MHz, DMSO-d6) δ 180.61 , 156.04 (dd, J = 246.5, 6.9 Hz), 153.88 , 152.34 (dd, J = 249.4, 8.6 Hz), 150.35 , 148.44 , 143.61 , 138.60 , 131.47 , 128.92 – 128.75 (m), 128.19 (d, J = 161.3 Hz), 126.05 , 121.94 (dd, J = 13.6, 3.6 Hz), 118.96 – 117.87 (m), 117.60 , 116.33 , 115.61 , 112.36 (dd, J = 22.6, 3.2 Hz), 79.03 , 53.42 , 48.08 , 43.72 , 42.58 , 28.09 , 16.87 , 12.64 . LC-MS (ESI); m/z $[M+H]^+$: Calcd. for $C_{32}H_{36}F_2N_5O_5S$, 640.2405. Found 640.2541.

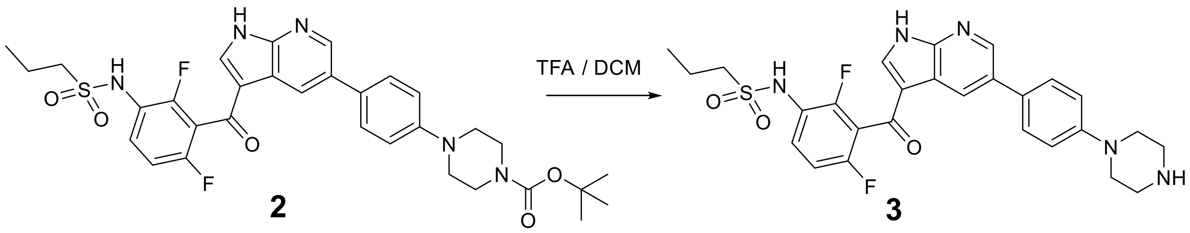

N-(2,4-difluoro-3-(5-(4-(piperazin-1-yl)phenyl)-1H-pyrrolo[2,3-b]pyridine-3-carbonyl)-phenyl)propane-1-sulfonamide (3). A solution of tert-butyl 4-[4-[3-[2,6-difluoro-3-(propylsulfonylamino)benzoyl]-1H-pyrrolo[2,3-b]pyridin-5-yl]phenyl]piperazine-1-carboxylate (**2**) (28.0 mg, 0.0438 mmol) in a mixture of DCM/TFA (3:1, 4 mL) was stirred for 1h at room temperature (by TLC no SM). The solvent was removed under vacuum and the residue was dried under high vacuum for 2h (23 mg of product, quantitative yield). Crude product was used in the next step without any further purification. LC-MS (ESI); m/z $[M+H]^+$: Calcd. for $C_{27}H_{28}F_2N_5O_3S$, 540.1880. Found 540.1949.

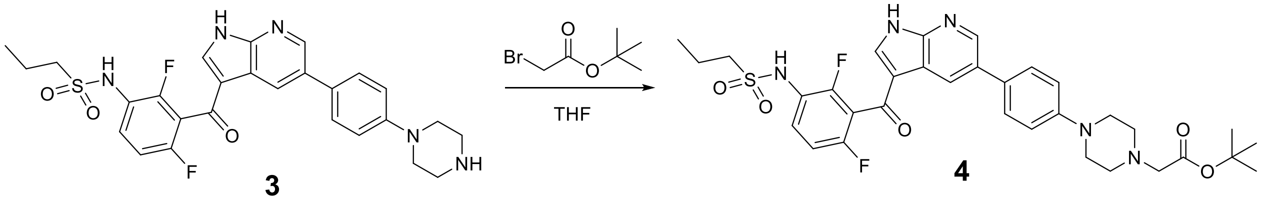

***tert*-Butyl 2-(4-(4-(3-(2,6-difluoro-3-(propylsulfonamido)benzoyl)-1H-pyrrolo[2,3-b]pyridin-5-yl)-phenyl) piperazin-1-yl)acetate (4)**. To a solution of N-[2,4-difluoro-3-[5-(4-piperazin-1-ylphenyl)-1H-pyrrolo[2,3-b]pyridine-3-carbonyl]-phenyl]propane-1-sulfonamide (**3**) (23.0 mg, 0.0426 mmol) and TEA

(0.0594 mL, 0.426 mmol) in DMF (1 ml) was added tert-butyl 2-bromoacetate (9.15 mg, 0.0469 mmol) and the resulting solution stirred for 3 h at rt. The reaction mixture was evaporated under vacuum. Crude product was purified by PTLC (DCM:MeOH:NH_4OH, 90:9:1, 2x) to give 19 mg of pure product (69% yield). ^{1}H NMR (500 MHz, DMSO-d6) δ 12.92 (bs, 1H), 9.76 (bs, 1H), 8.65 (t, J = 2.6 Hz, 1H), 8.55 (s, 1H), 8.18 (s, 1H), 7.76 – 7.42 (m, 3H), 7.28 (t, J = 8.3 Hz, 1H), 7.07 (d, J = 6.5 Hz, 2H), 3.33 (s, 2H), 3.30 – 3.16 (m, 4H), 3.16 – 3.04 (m, 2H), 2.81 – 2.55 (m, 4H), 1.84 – 1.67 (m, 2H), 1.43 (s, 9H), 0.96 (t, J = 7.2 Hz, 3H). ^{13}C NMR (151 MHz, DMSO-d6) δ 180.57 , 169.22 , 156.02 (dd, J = 246.3, 7.0 Hz), 152.33 (dd, J = 249.4, 8.6 Hz), 150.42 , 148.37 , 143.55 , 138.50 , 131.53 , 128.75 (d, J = 9.6 Hz), 128.17 , 127.58 , 125.93 , 121.92 (dd, J = 13.6, 3.6 Hz), 118.25 (t, J = 23.6 Hz), 117.58 , 115.80 , 115.58 , 112.33 (dd, J = 21.9, 3.0 Hz), 80.23 , 59.21 , 53.44 , 51.81 , 47.93 , 27.82 , 16.84 , 12.62 . LC-MS (ESI); m/z $[M+H]^+$: Calcd. for $C_{33}H_{38}F_2N_5O_5S$, 654.2561. Found 654.2675.

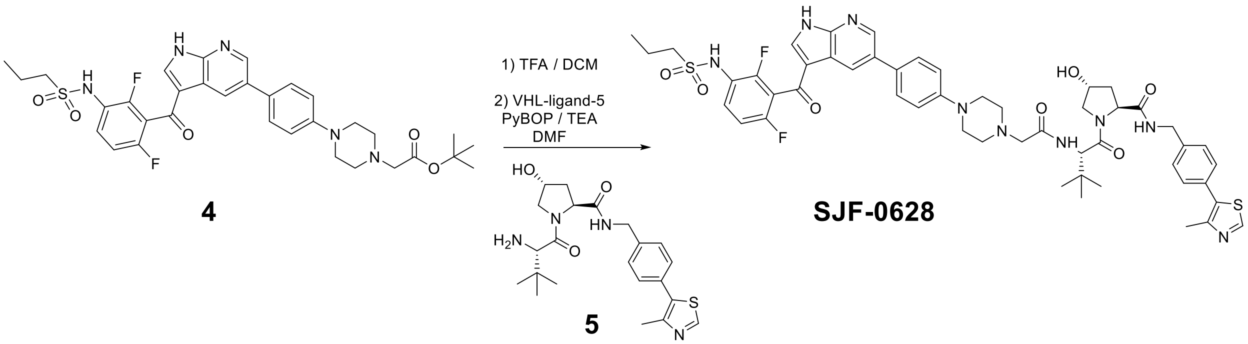

4

SJF-0628

5

(2S,4R)-1-((S)-2-(2-(4-(4-(3-(2,6-difluoro-3-(propylsulfonamido)benzoyl)-1H-pyrrolo[2,3-b]pyridin-5-yl)phenyl)piperazin-1-yl)acetamido)-3,3-dimethylbutanoyl)-4-hydroxy-N-(4-(4-methylthiazol-5-yl)benzyl)pyrrolidine-2-carboxamide (PROTAC SJF-0628). A solution oftert-butyl 2-(4-(4-(3-(2,6-difluoro-3-(propylsulfonamido)benzoyl)-1H-pyrrolo[2,3-b]pyridin-5-yl)phenyl)piperazin-1-yl)acetate (**4**) (20.0 mg, 0.0306 mmol) in a mixture of TFA (2 ml, 13.46 mmol) and DCM (2 ml) was stirred for 5 h. Then the solvent was removed under vacuum and crude product was dried under high vacuum for 1 h. Crude product was used in the next step without any further purification (18.3 mg, quantitative yield). LC-MS (ESI); m/z: $[M+H]^+$ Calcd. for $C_{29}H_{30}F_2N_5O_5S$, 598.1935. Found 598.1953. To a solution of crude product from above; 2-(4-(4-(3-(2,6-difluoro-3-(propylsulfonamido)benzoyl)-1H-pyrrolo[2,3-b]pyridin-5-yl)-phenyl) -piperazin-1-yl)acetic acid (18.3 mg, 0.0306 mmol) and (2S,4R)-1-[(2S)-2-amino-3,3-dimethyl-butanoyl]-4-hydroxy-N-[[4-(4-methylthiazol-5-yl)phenyl]methyl]pyrrolidine-2-carboxamide; hydro-chloride (**5**) (17.2 mg, 0.0367 mmol) in DMF (1 ml) was added TEA (0.106 mL, 0.762 mmol) and PyBOP (19.1 mg, 0.0367 mmol) at room temperature. The reaction mixture was stirred for 4 h at the same temperature. TLC (DCM:MeOH:NH_4OH, 90:9:1) shows no starting material (acid). The reaction mixture was evaporated to dryness under high vacuum. Crude product was diluted with EtOAc (10 mL) and washed with a saturated-aqueous solution of $NaHCO_3$ (2x5 mL), organic extract was dried (Na_2SO_4), and evaporated under vacuum. Crude product was purified by PTLC (DCM:MeOH:NH_4OH, 90:9:1, 2x) to give 20 mg of product (65% yield). ^{1}H NMR (500 MHz, DMSO-d6) δ 12.92 (bs, 1H), 9.76 (bs, 1H), 8.91 (s, 1H), 8.66 (s, 1H), 8.65 – 8.45 (m, 2H), 7.85 (d, J = 9.1 Hz, 1H), 7.74 – 7.52 (m, 3H), 7.49 – 7.32 (m, 4H), 7.28 (t, J = 8.6 Hz, 1H), 7.09 (d, J = 8.5 Hz, 2H), 5.16 (d, J = 3.0 Hz, 1H), 4.54 (d, J = 9.6 Hz, 1H), 4.52 – 4.32 (m, 3H), 4.26 (dd, J = 15.7, 5.4 Hz, 1H), 3.76 – 3.58 (m, 2H), 3.27 (s, 4H), 3.21 – 2.95 (m, 4H), 2.68 (s, 4H), 2.40 (s, 3H), 2.07 (dd, J = 12.9, 7.7 Hz, 1H),

1.92 (ddd, J = 13.1, 9.0, 4.6 Hz, 1H), 1.75 (h, J = 7.5 Hz, 2H), 0.97 (s, 9H), 0.96 (t, J = 7.4 Hz, 3H). ^{13}C NMR (126 MHz, DMSO-d6) δ 180.56 , 171.77 , 169.28 , 168.48 , 156.02 (dd, J = 246.6, 7.0 Hz), 152.37 (dd, J = 240.8, 8.8 Hz), 151.30 , 150.27 , 148.38 , 147.68 , 143.55 , 139.42 , 138.47 , 131.48 , 131.12 , 129.68 , 129.10 – 128.66 (m), 128.63 , 128.33 , 127.57 , 127.51 , 125.96 , 121.92 (dd, J = 13.7, 3.6 Hz), 118.60 – 117.95 (m), 117.57 , 115.84 , 115.59 , 112.32 (dd, J = 23.0, 2.7 Hz), 68.91 , 60.59 , 58.81 , 56.56 , 55.86 , 53.46, 52.75, 48.16 , 41.67 , 37.87 , 35.80 , 26.26 , 16.83 , 15.90 , 12.61. LC-MS (ESI); m/z $[M+H]^+$: Calcd. for $C_{51}H_{58}F_2N_9O_7S_2$, 1010.3868. Found 1010.4036.

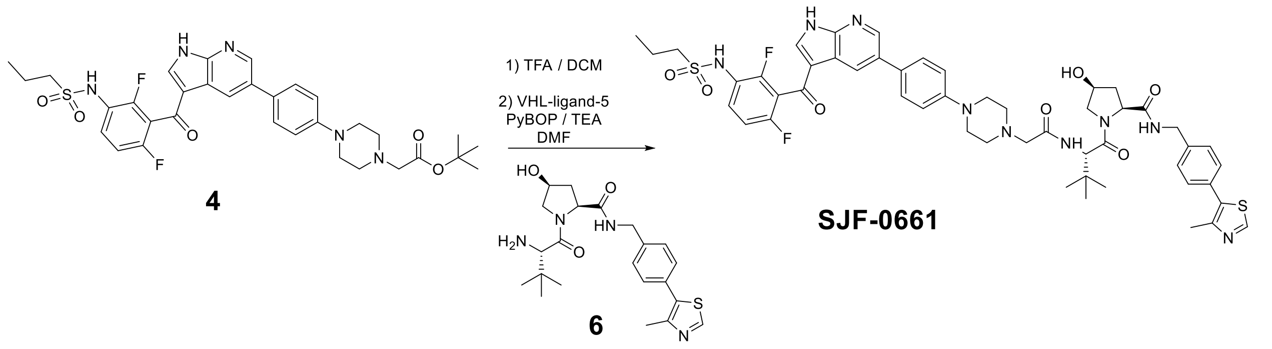

(2S,4S)-1-((S)-2-(2-(4-(4-(3-(2,6-difluoro-3-(propylsulfonamido)benzoyl)-1H-pyrrolo[2,3-b]pyridin-5-yl)phenyl)piperazin-1-yl)acetamido)-3,3-dimethylbutanoyl)-4-hydroxy-N-(4-(4-methylthiazol-5-yl)benzyl)pyrrolidine-2-carboxamide (SJF-0661). A solution of tert-butyl 2-(4-(4-(3-(2,6-difluoro-3-(propylsulfonamido)benzoyl)-1H-pyrrolo[2,3-b]pyridin-5-yl)phenyl)piperazin-1-yl)acetate (**4**) (20.0 mg, 0.0306 mmol) in a mixture of TFA (1 mL) and Dichloromethane (2 ml) was stirred for 5 h. Then the solvent was removed under vacuum and crude product was dried under high vacuum for 1 h. Crude product was used in the next step without any further purification (5.7 mg, quantitative yield). LC-MS (ESI); m/z: [M+H]+ Calcd. for $C_{29}H_{30}F_2N_5O_5S$, 598.1935. Found 598.1755. To a solution of crude product from above; 2-(4-(4-(3-(2,6-difluoro-3-(propylsulfonamido)benzoyl)-1H-pyrrolo[2,3-b]pyridine-5-yl)phenyl) piperazin-1-yl)acetic acid (5.70 mg, 0.00954 mmol) and (2S,4S)-1-((S)-2-amino-3,3-dimethylbutanoyl)-4-hydroxy-N-(4-(4-methylthiazol-5-yl)benzyl)pyrrolidine-2-carboxamide (**6**) (5.35 mg, 0.0124 mmol) in DMF (1 ml) was added TEA (0.1 mL, 0.762 mmol) and PyBOP (5.96 mg, 0.0114 mmol) at room temperature. The reaction mixture was stirred for 4 h at the same temperature. TLC (DCM:MeOH:NH4OH, 90:9:1) shows no starting material (acid). The reaction mixture was evaporated to dryness under high vacuum. Crude product was filtered over a silica-carbonate cartridge (100 mg) using DCM:MeOH (9:1) as a eluent and evaporated under vacuum.. Crude product was purified by PTLC (DCM:MeOH:NH_4OH, 90:9:1, 2x) to give 6.1 mg of product (63% yield). ^1H NMR (500 MHz, DMSO-d6) δ 12.93 (bs, 1H), 9.76 (bs, 1H), 8.93 (s, 1H), 8.69 (t, J = 5.8 Hz, 1H), 8.66 (s, 1H), 8.56 (bs, 1H), 8.18 (s, 1H), 7.81 (d, J = 8.6 Hz, 1H), 7.65 – 7.50 (m, 3H), 7.51 – 7.32 (m, 4H), 7.28 (t, J = 8.7 Hz, 1H), 7.09 (d, J = 8.2 Hz, 2H), 5.48 (d, J = 7.2 Hz, 1H), 4.49 (d, J = 9.2 Hz, 1H), 4.46 – 4.33 (m, 2H), 4.32 – 4.18 (m, 2H), 3.96 – 3.88 (m, 1H), 3.54 – 3.42 (m, 1H), 3.30 – 3.19 (m, 4H), 3.17 – 3.00 (m, 4H), 2.78 – 2.56 (m, 4H), 2.41 (s, 3H), 2.39 – 2.30 (m, 1H), 1.82 – 1.68 (m, 3H), 0.98 (s, 9H), 0.95 (t, 3H). ^{13}C NMR (151 MHz, DMSO-d6) δ 180.60 , 172.29 , 169.55 , 168.84 , 156.04 (dd, J = 246.5, 6.9 Hz), 152.38 (dd, J = 249.4, 8.3 Hz), 151.40 , 150.29 , 148.40 , 147.75 , 143.57 , 139.16 , 138.53 , 131.50 , 131.12 , 129.79 , 128.78 (d, J = 7.4 Hz), 128.70 , 128.36 , 127.54 , 125.99 , 121.93 (dd, J = 13.4, 3.6 Hz), 118.52 – 117.94 (m), 117.59 , 115.87 , 115.60 , 112.35 (dd, J = 23.0, 3.9

Hz), 69.09 , 60.50 , 58.62 , 56.01 , 55.63 , 53.46 , 52.70 , 48.17 , 41.83 , 36.90 , 35.21 , 26.25 , 16.85 , 15.93 , 12.63 . LC-MS (ESI); m/z $[M+H]^+$: Calcd. for $C_{51}H_{58}F_2N_9O_7S_2$, 1010.3868. Found 1010.3542.

3. Synthesis of PROTACs SJF-4604 and SJF-8090

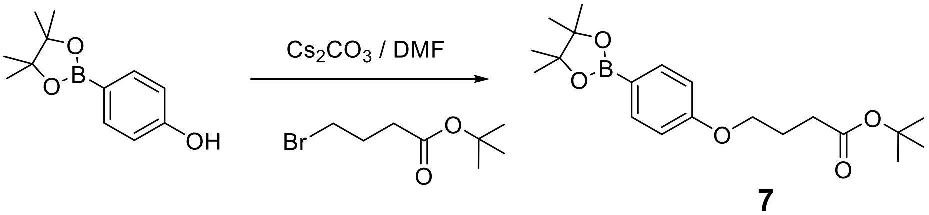

7

***tert*-Butyl 4-(4-(4,4,5,5-tetramethyl-1,3,2-dioxaborolan-2-yl)phenoxy)butanoate (7)**. To a mixture of 4-(4,4,5,5-tetramethyl-1,3,2-dioxaborolan-2-yl)phenol (209.12 mg, 0.95 mmol) and *tert*-butyl 4-bromobutanoate (212 mg, 0.95 mmol) in N,N-Dimethylformamide (2 mL) was added Cs_2CO_3 (402.47 mg, 1.24 mmol). Reaction mixture was heated at 65 °C for 12 h (overnight). By TLC small amounts of starting material (Hex:EtOAc, 7:3). Reaction mixture was diluted with EtOAc (10 mL), washed with water (4x10 mL), dried (Na_2SO_4) and evaporated under vacuum. Crude product was purified by flash CC (SiO_2-25g, Hex:EtOAc, gradient 9:1 to 4:6 in 15 min) to give 198 mg (57% yield) of product as an oil: ^{1}H NMR (500 MHz, DMSO-d6) δ 7.59 (d, J = 8.2 Hz, 2H), 6.91 (d, J = 7.9 Hz, 2H), 3.99 (t, J = 6.3 Hz, 2H), 2.35 (t, J = 7.3 Hz, 2H), 1.92 (p, J = 6.7 Hz, 2H), 1.39 (s, 9H), 1.27 (s, 12H). ^{13}C NMR (101 MHz, DMSO-d6) δ 172.25, 161.56, 136.66, 120.43, 114.37, 83.77, 80.12, 66.81, 31.72, 28.20, 25.12, 24.71. LC-MS (ESI); m/z $[M+Na]^+$: $C_{20}H_{31}BO_5Na$, 385.2162. Found 385.2194.

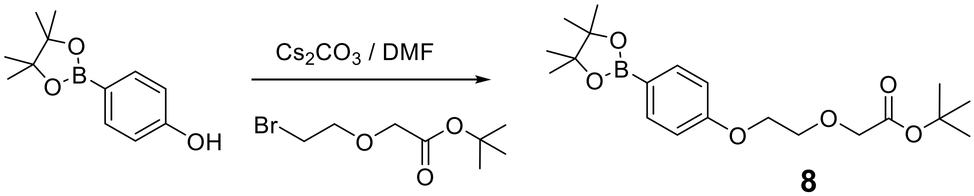

8

***tert*-Butyl 2-(2-(4-(4,4,5,5-tetramethyl-1,3,2-dioxaborolan-2-yl)phenoxy)ethoxy)acetate (8)**. To a mixture of 4-(4,4,5,5-tetramethyl-1,3,2-dioxaborolan-2-yl)phenol (100 mg, 0.45 mmol) and *tert*-butyl 2-(2-bromoethoxy)acetate (108.65 mg, 0.91 mmol) in N,N-Dimethylformamide (2 mL) was added Cs_2CO_3 (296 mg, 0.86 mmol). Reaction mixture was heated at 60 °C for 2 h. By TLC no starting material (less polar product, Hex:EtOAc, 7:3). Crude product was purified by flash CC (SiO_2-25g, Hex:EtOAc, 9:1 to 4:6 in 15 min) to give 134 mg of product as a waxy solid (70% yield). ^{1}H NMR (400 MHz, Chloroform-d) δ 7.74 (d, J = 8.5 Hz, 2H), 6.91 (d, J = 8.5 Hz, 2H), 4.19 (dd, J = 5.7, 3.8 Hz, 2H), 4.09 (s, 2H), 3.93 (dd, J = 5.8, 3.7 Hz, 2H), 1.48 (s, 9H), 1.33 (s, 12H). ^{13}C NMR (151 MHz, cdcl3) δ 169.71, 161.35, 136.62, 121.07, 114.05, 83.70, 81.87, 69.94, 69.36, 67.37, 28.26, 25.01. LC-MS (ESI); m/z: $[M+Na]^+$ Calcd. for $C_{20}H_{31}BO_6Na$, 401.2111. Found 401.2102.

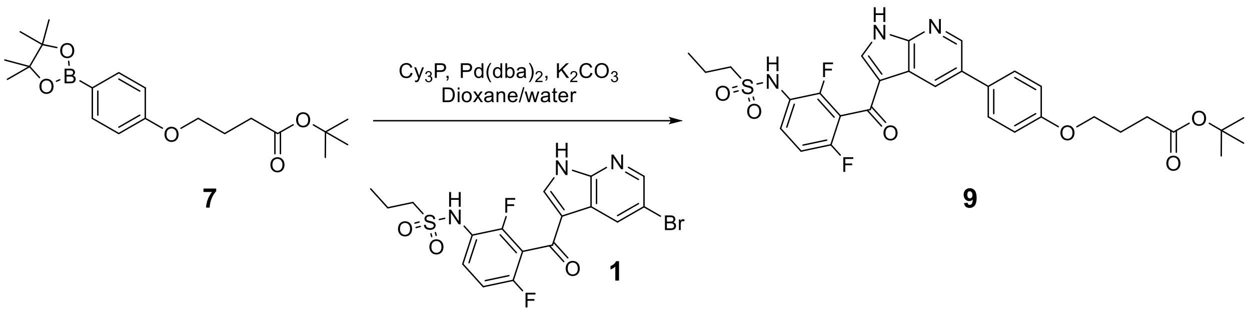

***tert*-Butyl 4-(4-(3-(2,6-difluoro-3-(propylsulfonamido)benzoyl)-1H-pyrrolo[2,3-b]pyridin-5-yl)phenoxy)- butanoate (9)**. To a solution of *tert*-butyl 4-[4-(4,4,5,5-tetramethyl-1,3,2-dioxaborolan-2-yl)phenoxy] -butanoate (65 mg, 0.18 mmol) in Dioxane (3 ml) in a microwave vial was added N-[3-(5-bromo-1H-pyrrolo[2,3-b]pyridine-3-carbonyl)-2,4-difluoro-phenyl]propane-1-sulfonamide (82.2 mg, 0.18 mmol). Then the reaction mixture was de-gassed under vacuum and purged with argon (5x). Then Tricyclohexylphosphine (5.03 mg, 0.0179 mmol) and Pd(dba)2 (5.16 mg, 0.01 mmol) were added into and the vial was caped and sealed under a stream of argon, then the reaction mixture was heated at 100 °C in a microwave reactor for 2 h. By TLC no starting material (Hex:EtOAc, 3:7), the reaction mixture was filtered in vacuo over a celite pad. Filtrate was poured onto an aqueous saturated solution of NaCl (20 mL) and the product was extracted with EtOAc (2x20 mL). The EtOAc layers were combined, dried (Na_2SO_4) and concentrated in vacuo. The crude material was diluted in DCM and purified by flash chromatography (SiO_2-12g, Hexane:EtOAc, 8:2 to 100% EtOAc in 15 min) to give 49 mg (40%) of product as a pale solid. ^{1}H NMR (500 MHz, DMSO-d6) δ 12.93 (s, 1H), 9.74 (s, 1H), 8.64 (d, J = 2.0 Hz, 1H), 8.55 (bs, 1H), 8.19 (s, 1H), 7.65 (d, J = 8.5 Hz, 2H), 7.57 (td, J = 9.0, 5.6 Hz, 1H), 7.26 (t, J = 8.6 Hz, 1H), 7.05 (d, J = 8.6 Hz, 2H), 4.03 (t, J = 6.3 Hz, 2H), 3.15 – 3.06 (m, 2H), 2.38 (t, J = 7.3 Hz, 2H), 1.95 (p, J = 6.8 Hz, 2H), 1.78 – 1.67 (m, 2H), 1.39 (s, 9H), 0.94 (t, J = 7.4 Hz, 3H). ^{13}C NMR (126 MHz, DMSO-d6) δ 181.02, 172.32, 158.70, 157.46, 157.41, 156.46 (d, J = 240.0 Hz), 152.76 (dd, J = 249.2, 8.3 Hz), 148.97, 144.17, 131.74, 130.92, 129.18 (d, J = 12.4 Hz), 128.71, 126.86, 122.34 (d, J = 13.2 Hz), 119.81 – 117.27 (m), 117.96, 116.05, 115.59, 112.76 (d, J = 27.7 Hz). 80.13, 67.09, 53.89, 31.80, 28.22, 24.81, 17.27, 13.05. LC-MS (ESI); m/z: $[M+H]^+$ Calcd. for $C_{31}H_{34}F_2N_3O_6S$, 614.2136. Found 614.2281.

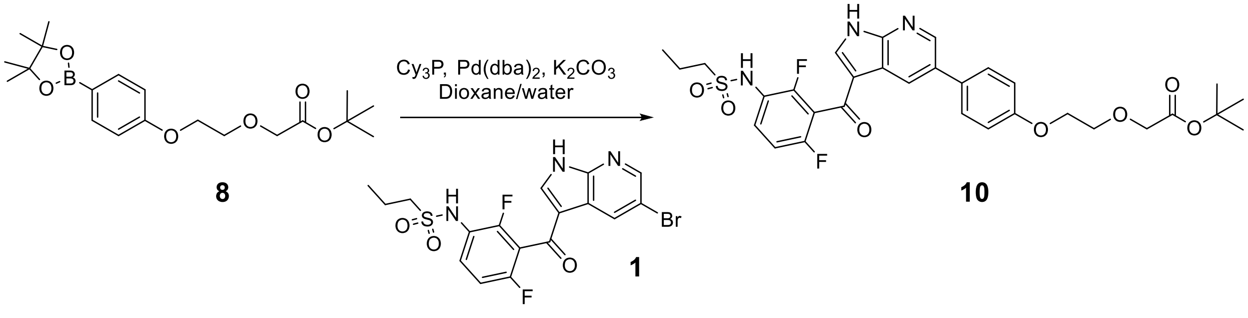

***tert*-Butyl 2-(2-(4-(3-(2,6-difluoro-3-(propylsulfonamido)benzoyl)-1H-pyrrolo[2,3-b]pyridin-5-yl)-phenoxy)ethoxy)acetate (10**). To a solution of *tert*-butyl 2-[2-[4-(4,4,5,5-tetramethyl-1,3,2-dioxaborolan-2-yl)phenoxy]ethoxy]acetate (54 mg, 0.14 mmol) and N-[3-(5-bromo-1H-pyrrolo[2,3-b]pyridine-3-carbonyl)-2,4-difluoro-phenyl]propane-1-sulfonamide (72 mg, 0.16 mmol) in Dioxane (6 ml) was degassed under vacuum and purged with argon (5x). Then K_2CO_3 (210 mg, 1.52 mmol) and Water (3 ml) was added. The reaction mixture was degassed again under vacuum and purged with

argon (5x). Then Tricyclohexylphosphine (14.27 mg, 0.05 mmol) and Pd(dba)$_2$ (14.6 mg, 0.025 mmol) were added into and degassed again under vacuum and purged with argon (5x). The reaction mixture was heated with vigorous stirring at 80 °C and stirred for 12 h. By TLC (DCM:MeOH:NH_4OH, 90:9:1, 3X) full conversion (SM and product have a very similar r.f.). The reaction mixture was diluted with EtOAc (50 mL) and filtered over a celite pad. Filtrate was washed with an aqueous saturated solution of NaCl (30 mL) and product was extracted with EtOAc (2x50 mL). EtOAc layers combined, dried (Na_2SO_4) and concentrated under vacuo. Crude product was purified by flash CC (SiO_2-12g, Hex:EtOAc, 1:9 to 100% in 15 min) to give 40 mg of product (40% yield). ^{1}H NMR (400 MHz, DMSO-d6) δ 12.95 (bs, 1H), 9.77 (bs, 1H), 8.67 (s, 1H), 8.58 (s, 1H), 8.21 (s, 1H), 7.68 (d, J = 8.3 Hz, 2H), 7.59 (q, J = 8.8 Hz, 1H), 7.28 (t, J = 8.7 Hz, 1H), 7.09 (d, J = 8.3 Hz, 2H), 4.18 (dd, J = 5.6, 3.3 Hz, 2H), 4.08 (s, 2H), 3.84 (dd, J = 5.5, 3.4 Hz, 2H), 3.19 – 3.06 (m, 2H), 1.74 (h, J = 7.5 Hz, 2H), 1.43 (s, 9H), 0.96 (t, J = 7.4 Hz, 3H). ^{13}C NMR (151 MHz, DMSO-d6) δ 180.61 , 169.33 , 158.18 , 156.05 (d, J = 246.4 Hz), 152.31 (d, J = 249.5 Hz), 148.56 , 143.75 , 138.66 , 131.30 , 130.58 , 128.76 (d, J = 9.8 Hz), 128.29 , 126.47 , 121.94 (dd, J = 13.6, 3.6 Hz), 118.23 (dd, J = 24.6, 22.3 Hz), 117.54 , 115.63 , 115.17 , 112.34 (dd, J = 22.8, 3.9 Hz), 80.76 , 69.07 , 68.19 , 67.18 , 53.45 , 27.77 , 16.85 , 12.62. LC-MS (ESI); m/z: $[M+H]^+$ Calcd. for $C_{31}H_{34}F_2N_3O_7S$, 630.2085. Found 630.5872.

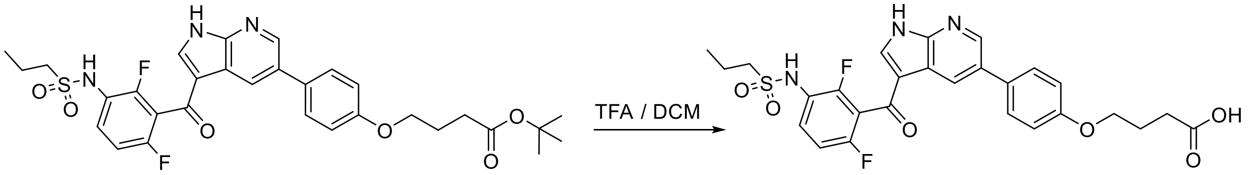

9 **11**

4-(4-(3-(2,6-Difluoro-3-(propylsulfonamido)benzoyl)-1H-pyrrolo[2,3-b]pyridin-5-yl)phenoxy)butanoic acid (11). A solution of *tert*-butyl 4-[4-[3-[2,6-difluoro-3-(propylsulfonylamino)benzoyl] -1H-pyrrolo[2,3-b]pyridin-5-yl]phenoxy]butanoate (16 mg, 0.03 mmol) in a mixture of TFA (1 ml, 13.46 mmol) and Dichloromethane (2 ml) was stirred at room temperature for 2 h. Then the solvent was removed under vacuum and crude product was dried under high vacuum for 2 h. Crude product was used in the next step without any further purification (14.5 mg, quantitative yield). LC-MS (ESI); m/z: $[M+H]^+$ Calcd. for $C_{27}H_{26}F_2N_3O_6S$, 558.1510. Found 558.1603.

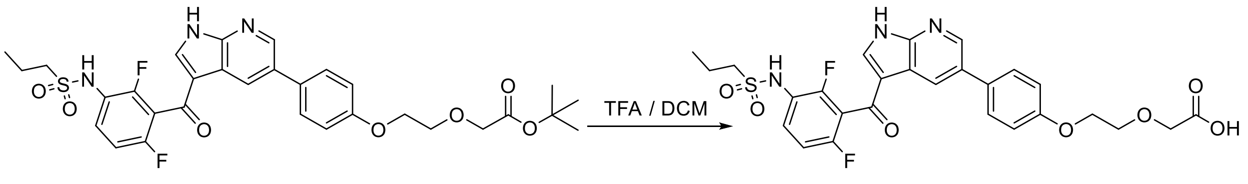

10 **12**

2-(2-(4-(3-(2,6-Difluoro-3-(propylsulfonamido)benzoyl)-1H-pyrrolo[2,3-b]pyridin-5-yl)phenoxy)ethoxy) -acetic acid (12). A solution of *tert*-butyl 2-[2-[4-[3-[2,6-difluoro-3-(propylsulfonylamino)benzoyl]-1H-pyrrolo[2,3-b]-pyridin-5-yl]phenoxy]ethoxy]acetate (8 mg, 0.013 mmol) in a mixture of TFA (1 ml, 13.46 mmol) and Dichloromethane (2 ml) was stirred at room temperature for 2 h. Then the solvent was removed under vacuum and crude product was dried under

high vacuum for 2 h. Crude product was used in the next step without any further purification (7.2 mg, quantitative yield). LC-MS (ESI); m/z: $[M+H]^+$ Calcd. for $C_{27}H_{26}F_2N_3O_7S$, 574.1459. Found 574.3837.

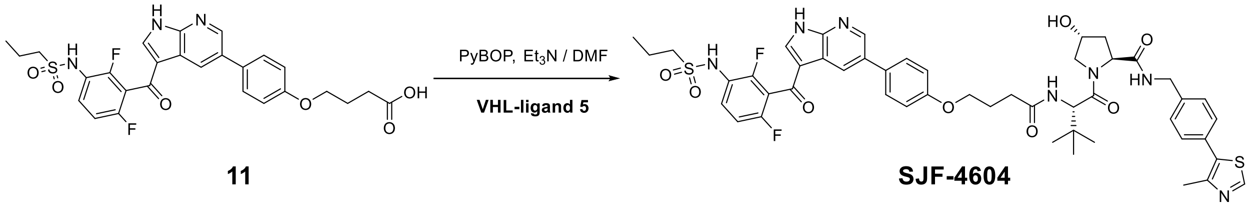

11 **SJF-4604**

(2S,4R)-1-((S)-2-(4-(4-(3-(2,6-Difluoro-3-(propylsulfonamido)benzoyl)-1H-pyrrolo[2,3-b]pyridin-5-yl)phenoxy)butanamido)-3,3-dimethylbutanoyl)-4-hydroxy-N-(4-(4-methylthiazol-5-yl)benzyl) pyrrolidine-2-carboxamide (PROTAC SJF-4604). To a solution of crude product from **11**; 4-[4-[3-[2,6-difluoro-3-(propylsulfonylamino)-benzoyl]-1H-pyrrolo[2,3-b]pyridin-5-yl]phen- oxy]butanoic acid (14.5 mg, 0.026 mmol) and **VHL-ligand 5** (2S,4R)-1-[(2S)-2-amino-3,3-dimethyl-butanoyl]-4-hydroxy-N-[[4-(4-methylthiazol-5-yl)-phenyl]methyl] -pyrrolidine-2-carboxamide;hydrochloride (13 mg, 0.029 mmol) in DMF(2 ml) was added TEA (0.1 ml, 0.72 mmol) and PyBOP (14.9 mg, 0.029 mmol) at room temperature. The reaction mixture was stirred for 4 h at the same temperature. TLC (DCM:MeOH:NH_4OH, 90:9:1) shows no starting materials. The DMF was removed under high vacuum. Crude product was filtered over a silica-carbonate cartridge using DCM:MeOH (9:1) as a eluent. Filtrate was evaporated under vacuum and crude product was purified by PTLC (DCM:MeOH:NH_4OH, 90:9:1, 2x) to give 14 mg of product (55% yield). ^1H NMR (400 MHz, DMSO-d6) δ 12.89 (bs, 1H), 9.72 (bs, 1H), 8.97 (s, 1H), 8.66 (d, J = 2.1 Hz, 1H), 8.58 (t, J = 5.7 Hz, 2H), 8.20 (s, 1H), 8.01 (d, J = 9.3 Hz, 1H), 7.66 (d, J = 8.3 Hz, 2H), 7.59 (td, J = 9.0, 5.8 Hz, 1H), 7.43 (d, J = 8.2 Hz, 2H), 7.38 (d, J = 8.2 Hz, 2H), 7.28 (t, J = 8.5 Hz, 1H), 7.07 (d, J = 8.7 Hz, 2H), 5.15 (d, J = 3.3 Hz, 1H), 4.58 (d, J = 9.3 Hz, 1H), 4.50 – 4.40 (m, 2H), 4.36 (bs, 1H), 4.22 (dd, J = 15.8, 5.3 Hz, 1H), 4.03 (t, J = 6.1 Hz, 2H), 3.76 – 3.61 (m, 2H), 3.17 – 3.05 (m, 2H), 2.44 (s, 3H), 2.49 – 2.31 (m, 2H), 2.13 – 1.85 (m, 4H), 1.74 (dq, J = 14.9, 7.4 Hz, 2H), 0.96 (s, 9H), 0.95 (t, 3H). ^{13}C NMR (151 MHz, DMSO-d6) δ 181.03, 172.39, 172.03, 170.09, 156.43 (dd, J = 246.4, 6.9 Hz), 158.76, 152.75 (dd, J = 249.5, 8.5 Hz), 151.86, 148.95, 148.13, 144.17, 139.06, 131.76, 131.59, 130.81, 130.06, 129.18 (d, J = 14.4 Hz), 129.06, 128.69, 127.85, 126.86, 122.39 (dd, J = 13.8, 3.2 Hz), 118.,94 – 118.29 (m), 117.95, 116.04, 115.61, 112.75 (dd, J = 22.5, 3.3 Hz), 69.33, 67.55, 59.15, 56.90, 56.84, 53.87, 42.08, 38.40, 35.68, 31.74, 26.83, 25.48, 17.27, 16.38, 13.04. LC-MS (ESI); m/z $[M+H]^+$: Calcd. for $C_{49}H_{54}F_2N_7O_8S_2$, 970.3443. Found 970.3176.

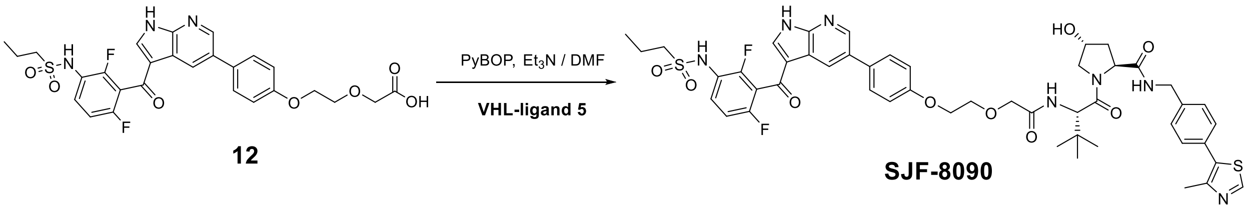

(2S,4R)-1-((S)-2-(2-(2-(4-(3-(2,6-Difluoro-3-(propylsulfonamido)benzoyl)-1H-pyrrolo[2,3-b]pyridin-5-yl)phenoxy)ethoxy)acetamido)-3,3-dimethylbutanoyl)-4-hydroxy-N-(4-(4-methylthiazol-5-yl)benzyl)- pyrrolidine-2-carboxamide (PROTAC SJF-8090). To a solution of crude product from **12** ; 2-[2-[4-[3-[2,6-difluoro-3- (propylsulfonylamino)benzoyl]-1H-pyrrolo -[2,3-b]pyridin-5-yl]phenoxy]ethoxy]acetic acid (7.28 mg, 0.01 mmol) and **VHL-ligand 5** (2S,4R)-1-[(2S)-2-amino-3,3-dimethyl-butanoyl]-4-hydroxy-N-[[4-(4-methylthiazol-5-yl)-phenyl]methyl]pyrrolidine-2-carboxamide; hydrochloride (8.89 mg, 0.02 mmol) in DMF(2 ml) was added TEA (0.05 ml, 0.34 mmol) and PyBOP (7.93 mg, 0.02 mmol) at room temperature. The reaction mixture was stirred for 2 h at the same temperature. TLC (DCM:MeOH:NH_4OH, 90:9:1) shows no starting materials. Reaction mixture was diluted with EtOAc (10 mL), washed with water (3x10 mL), dried (Na_2SO_4) and evaporated under vacuum to give 1 mg of crude product (product is partially soluble in water). Additional water extractions with EtOAc (5x30 mL) were performed. Organic extracts combined, dried (Na_2SO_4), and evaporated under high vacuum. Crude product was purified by PTLC (DCM:MeOH:NH_4OH, 90:9:1, 2x) to give 5 mg of product (40 % total yield).^{1}H NMR (500 MHz, DMSO-d6) δ 12.92 (bs, 1H), 9.73 (bs, 1H), 8.88 (s, 1H), 8.69 – 8.55 (m, 2H), 8.54 (bs, 1H), 8.19 (s, 1H), 7.67 – 7.54 (m, 3H), 7.53 (d, J = 9.6 Hz, 1H), 7.42 (d, J = 8.1 Hz, 2H), 7.36 (d, J = 8.1 Hz, 2H), 7.28 (t, J = 8.8 Hz, 1H), 7.16 (d, J = 8.7 Hz, 2H), 5.16 (d, J = 3.5 Hz, 1H), 4.62 (d, J = 9.6 Hz, 1H), 4.52 – 4.33 (m, 3H), 4.32 – 4.13 (m, 3H), 4.08 (s, 2H), 3.88 (t, J = 4.3 Hz, 2H), 3.76 – 3.57 (m, 2H), 3.19 – 3.03 (m, 2H), 2.37 (s, 3H), 2.11 – 1.99 (m, 1H), 1.97 – 1.84 (m, 1H), 1.81 – 1.64 (m, 2H), 0.97 (s, 9H), 0.96 (t, 3H). ^{13}C NMR (151 MHz, DMSO-d6) δ 181.02, 172.25, 169.56, 168.95, 158.66, 151.69, 156.43 (dd, J = 246.3, 7.3 Hz), 152.75 (dd, J = 249.6, 8.6 Hz), 148.95, 148.09, 144.13, 139.84, 139.24 – 138.83 (m), 131.68, 131.50, 131.04, 130.07, 129.30, 129.08, 128.65, 127.87, 126.90, 122.37 (d, J = 15.4 Hz), 118.71 (d, J = 23.4 Hz), 117.93, 116.05, 115.69, 112.75 (dd, J = 23.3, 3.7 Hz), 70.02, 69.96, 69.33, 67.45, 59.18, 57.04, 56.15, 53.86, 42.14, 38.34, 36.28, 26.66, 17.27, 16.29, 13.04. LC-MS (ESI); m/z $[M+H]^+$: Calcd. for $C_{49}H_{54}F_2N_7O_9S_2$, 986.3392. Found 986.3481.

Supplementary References

1. Bollag G*, et al.* Clinical efficacy of a RAF inhibitor needs broad target blockade in BRAF-mutant melanoma. *Nature* **467**, 596-599 (2010).

2. Hildbrand S, Mair HJ, Radinov RN, Ren Y, Wright JA. Process for the manufacture of pharmaceutically active compounds. *Patent No. US 8,329,724 B2* (2012).

3. Maniaci C*, et al.* Homo-PROTACs: bivalent small-molecule dimerizers of the VHL E3 ubiquitin ligase to induce self-degradation. *Nat Commun* **8**, 830 (2017).
